# Function and regulation annotation of up‐regulated long non‐coding RNA *LINC01234* in gastric cancer

**DOI:** 10.1002/jcla.23210

**Published:** 2020-02-03

**Authors:** Yinyin Zhu, Cong Luo, Arshad Ali Korakkandan, Yislam Hadi Ahmed Fatma, Yang Tao, Tianfei Yi, Shiyun Hu, Qi Liao

**Affiliations:** ^1^ Department of Preventative Medicine Zhejiang Provincial Key Laboratory of Pathological and Physiological Technology Medical School of Ningbo University Ningbo China; ^2^ Department of Abdominal Oncology Zhejiang Cancer Hospital Hangzhou China; ^3^ Ningbo Yinzhou People's Hospital Ningbo China

**Keywords:** gastric cancer, *LINC01234*, long non‐coding RNA, regulatory network

## Abstract

**Background:**

Accumulated evidences indicate that long non‐coding RNAs (lncRNAs) participate in many biological mechanisms. Moreover, it acts as an essential regulator in various human diseases such as gastric cancer (GC). Nevertheless, the comprehensive regulatory roles and clinical significance of most lncRNAs in GC are not fully understood.

**Methods:**

In this research, our aim was to investigate the underlying mechanism of lncRNA *LINC01234* in GC. Firstly, the usage of qRT‐PCR helped to establish expression pattern of *LINC01234* in GC tissues. Following this, appropriate statistical tests were applied to analyze the relation between expression level and clinicopathological factors. Ultimately, potential functions and regulatory network of *LINC01234* were concluded via GSEA and a series of bioinformatics tools or databases, respectively.

**Results:**

Consequently, at the end of research we found *LINC01234* is up‐regulated in GC tissues in comparison with adjacent normal tissues. Furthermore, its expression level is correlated with differentiation of patients with GC. It is also important to highlight bioinformatics analysis revealed that *LINC01234 is* involved in cancer‐associated pathways such as cell cycle and mismatch repair. Also, regulatory network of *LINC01234* presented a probability in the involvement of tumorigenesis through regulating cancer‐associated genes.

**Conclusion:**

Overall, our results suggested that *LINC01234* may play a crucial role in GC.

**Abbreviations**
AUC: area under the ROC curve
EMT: epithelial‐to‐mesenchymal transition
ESCC: esophageal squamous cell carcinoma
FDR: false discovery rate
FPKM: Fragments per Kilobase of transcript per Million fragments mapped
GC: gastric cancer
GSEA: gene set enrichment analysis
lncRNA: long non‐coding RNA
qRT‐PCR: real‐time quantitative reverse transcription‐polymerase chain reaction
ROC: receiver operating characteristic
TCGA: the cancer genome atlas

## 1 INTRODUCTION

Gastric cancer (GC) is a complex disease caused by accumulation of both genetic and epigenetic factors and imposes a considerable global health burden.^1^ In fact, in 2016, there were 1.2 million cases of GC with 834 000 deaths worldwide and 18.3 million DALYs (disability‐adjusted life years).^2^ Even though scientists are making great efforts and there is a steady decline in GC incidence and mortality rates, it is still hard to diagnose GC patients at early stage. To illustrate, this means most GC patients are missing their opportunity for radical gastrectomy, which is currently the best way to cure GC when diagnosed.^3^, ^4^ Additionally, many patients have a significant risk of metastasis and low survival time even after curative resection. Thus, it is vital to identify new effective biomarkers and therapeutic target agents for the treatment and early diagnosis of GC.

Long non‐coding RNAs (lncRNAs) are a kind of non‐coding RNAs (lacking the ability of encoding protein) with a length larger than 200 nt. Even though the same as mRNAs, lncRNAs are also transcribed out of DNA by RNA polymerase II; they were initially thought to be a noise in transcriptome when first found.^5^ Despite this, accumulated studies established that lncRNAs function as regulators of gene expression, stability, and location at the epigenetic, transcriptional, and post‐transcriptional levels.^6^, ^7^ Thus, aberrance of lncRNA expression is involved in numerous biological processes such as cell cycle, cell differentiation, proliferation, apoptosis, metastasis, invasion, and migration in several kinds of cancer including GC.^8^, ^9^ Also, some lncRNAs can indeed be used as a biomarker for the diagnosis and prognosis in many kinds of cancers such as breast cancer^10^ and GC.^11^ For example, *LINC1006* 12 was found to be a novel biomarker for GC previously. Above all, lncRNAs are important in both the initiation and development of GC.

Over the last decades, numerous experimental researches have identified several lncRNAs that play crucial role in GC such as imprinted maternally expressed transcript (*H19*),^13^ small nucleolar RNA host gene 5 (*SNHG5*),^14^ homeobox transcript antisense RNA (*HOTAIR*),^15^ AGAP2 antisense RNA 1 (*AGAP2‐AS1*),^16^ and Pvt1 oncogene (*PVT1*).^17^ Yet they were only a tip of iceberg, there remain a large number of lncRNAs with unknown functions and regulation mechanism in GC. Due to the advances of sequencing technology, more and more high‐throughput data of transcriptome in GC were carried out. The Cancer Genome Atlas (TCGA) collects sequencing data of genome, transcriptome, and epigenome from many patients with various kinds of cancer including stomach adenocarcinoma (STAD). It provides an opportunity to dig out unknown genes especially for those lncRNAs in GC.

In this research, we first analyzed gene expression profiles of STAD patients in TCGA and found a number of lncRNAs differently expressed in cancerous tissues compared with adjacent non‐cancerous tissues. Then, we verified one of the up‐regulated lncRNA, *LINC01234*, in GC tissues compared with adjacent non‐cancerous tissues by real‐time quantitative reverse transcription‐polymerase chain reaction (qRT‐PCR). Also, the association between expression level of *LINC01234* and clinicopathological factors was analyzed. Subsequently, we annotated the functions of *LINC01234* using Gene Set Enrichment Analysis (GSEA) method and constructed the *LINC01234* regulatory network to well interpret the regulation mechanism of *LINC01234* in GC.

## 2 MATERIALS AND METHODS

### 2.1 Differently expression analysis of lncRNAs in STAD from TCGA

Fragments per Kilobase of transcript per Million fragments mapped (FPKM) expression profiles and clinical information of STAD patients were downloaded from TCGA website (https://portal.gdc.cancer.gov/). There are 375 cancerous samples and 32 adjacent normal samples (Table S1). Long intergenic non‐coding RNA (lincRNA) and antisense RNAs were selected as lncRNAs and were analyzed by* t* test. False discovery rate (FDR) method was used to correct *P* values. Those with FDR < 0.05 and fold change larger than 1.5 were considered to be as differently expressed lncRNAs.

### 2.2 Collection of GC samples and patients' clinical information

Paired cancerous and adjacent normal tissues of 83 GC patients were collected during surgery in the span of 2010 to 2015 at Zhejiang Cancer Hospital. The adjacent normal tissues were defined as those tissues located 5 cm away from the edge of the tumor. All the samples with a size of around 0.1 cm^3^ were immediately preserved in RNA fixer (BioTeke) and stored at −80°C until use. For each GC patient, the clinical information consisted of age, gender, invasion depth, differentiation, lymphatic metastasis, distal metastasis, and TNM stage. It is important to state no patient had undergone preoperative radiotherapy or chemotherapy. Also, each patient had handed over a written consent with a signed name indicating they are willing to participate in this research and the ethics committee of Ningbo University approved for this investigation.

### 2.3 Total RNA extraction and qRT‐PCR

The methods for total RNA preparation and qRT‐PCR were analogous to our previous study.^18^ For instance, we extracted the total RNA using TRIzol reagent (Thermo Fisher Scientific) from each cancer tissue and adjacent normal tissue. From here, we were able to detect total RNA by using a protein‐nucleic acid spectrophotometer according to A260/280 ratio. Hereafter, 2 μg RNA was reverse‐transcribed into cDNA with GoTaq qPCR Master Mix (Promega) and the process of qRT‐PCR was performed on LightCycler 480 (Roche). The sequences of PCR primers for β‐actin were 5′‐CATGTACGTTGCTATCCAGGC‐3′ (forward) and 5′‐CTCCTTAATGTCACGCACGAT‐3′ (reverse). On the other hand, the sequences of PCR primers for *LINC01234* were 5‐TCTACTAGAGCCTCCAGAAGG‐3′ (forward) and 5‐CTACTCTTCACGCAGAGGA‐3′ (reverse). Importantly, the conditions of thermal cycling were as follows: predegeneration at 95°C in 10 minutes, after which 45 cycles at 95°C for 15 seconds, 55°C for 30 seconds, and 72°C for 30 seconds. The expression level of *LINC01234* was calculated using the ΔCt method with β‐actin expression value as control, which was calculated by subtracting the Ct values of β‐actin from the Ct values of *LINC01234*. Then, ΔΔCt of *LINC01234* was calculated by subtracting ΔCt of adjacent non‐cancerous tissue from that of the paired cancer tissue. At last, the fold change of *LINC01234* was calculated by the equation 2^−ΔΔCt^. All results were described as the expression of mean ± standard deviation of three independent experiments.

### 2.4 Gene set enrichment analysis of *LINC01234*

By using the median expression level of *LINC01234* as cutoff, STAD patients were divided into two groups: with low expression and high expression of *LINC01234,* respectively. Subsequently, FPKM expression profiles for STAD patients and group labels of samples were put into GSEA software.^19^ Gene Ontology (GO) Biological Process (BP) term and KEGG pathway datasets were selected to calculate the enriched functions and pathways associated with *LINC01234*. Adjusted *P*‐value < .05 was considered to be statistically significant.

### 2.5 Construction of *LINC01234* regulatory network

#### 2.5.1 TF‐LINC01234 regulation

We downloaded the genomic location of peaks of transcription factor (TF) from Cistrome databases,^20^ which re‐calculated ChIP‐seq datasets for TF and histone modification from GEO database.^21^ Next, we compared the chromosome position of these binding regions with that of *LINC01234* *,* only those with the binding sites locating promoters of *LINC01234* were considered as TF‐*LINC01234* regulation relationships. Then, TFs were filtered by differently expressed protein‐coding genes in GC identified from STAD expression profiles from TCGA by using *t* test. False discovery rate (FDR) method was used to correct *P*‐values for multiple comparisons, and .05 was set as a cutoff.

#### 2.5.2 miRNA‐LINC01234 interactions

The conclusion of miRNA‐*LINC01234* interactions was established upon reliable miRNA target prediction tool known as miRanda set on default parameters.^22^ Due to the up‐regulation of *LINC01234* in GC, only those miRNAs down‐regulated in GC were obtained according to miRCancer database.^23^

#### 2.5.3 RBP‐LINC01234 interactions

Likewise, prediction of RBP*‐LINC01234* interactions was set by utilizing a model called lncPro^24^ using sequence information downloaded from UniProt.^25^ Afterward, RBP was also filtered by removing non‐differently expressed protein‐coding genes in STAD from TCGA.

### 2.6 Statistical analysis

IBM SPSS 21.0 software (SPSS) and R 3.3.3 were the two software used to perform statistical analysis. Comparison of “expression values” among three or more groups was analyzed by one‐way analyses of variance (ANOVAs), while that between two groups was performed by Student's *t* test. Statistical differences were set at *P < .05, **P < .01, and ***P < .001. *P* < .05 was set to analyze the statistical significances.

## 3 RESULTS

### 3.1 Experimental verification of *LINC01234* up‐regulation in GC tissues

**Figure 1:**
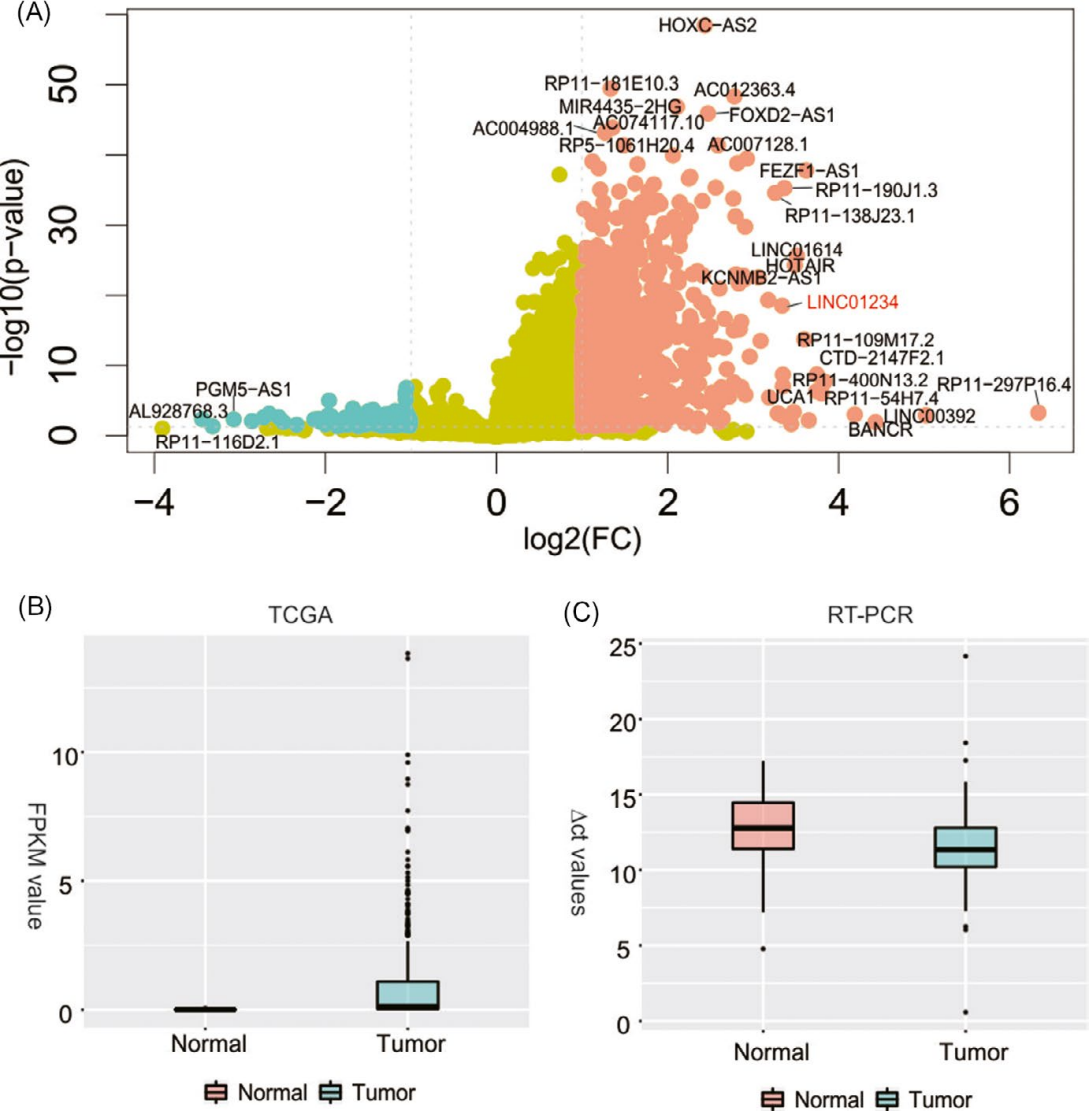
A, Differently expressed lncRNAs in STAD. B, Expression level (FPKM) of *LINC01234* in STAD tissues compared with adjacent normal tissues. C, Expression level (△Ct value) of *LINC01234* in GC tissues compared with adjacent normal tissues

Firstly, we downloaded the expression profiles of STAD from TCGA and investigated the differently expressed lncRNAs. Consequently, 1016 up‐regulated and 140 down‐regulated lncRNAs in GC compared with non‐cancer tissues were found (Figure 1A, Table S2). Among them, one of up‐regulated lncRNAs, *LINC01234*, was selected to study deeply because of poor knowledge of it in GC. We then verified the disorder expression pattern of *LINC01234* using qRT‐PCR in 83 GC tissues and adjacent normal tissues (Figure 1B). Hence, by comparing the adjacent non‐cancerous tissues, it is concluded that *LINC01234* is strictly up‐regulated in 61 of 83 GC tissues (73.5%, Figure 1C, *P* < .001).

### 3.2 Association analysis between expression level of *LINC01234* and clinicopathological factors in GC patients

**Figure 2:**
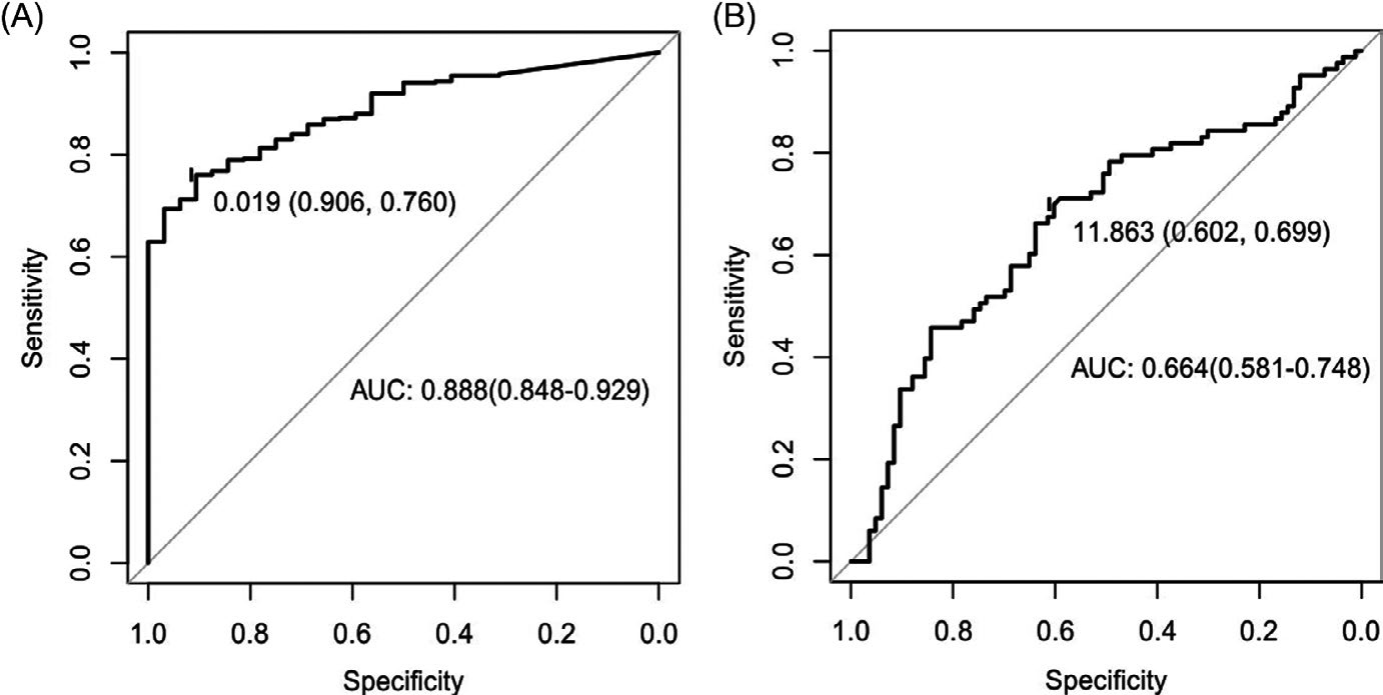
Diagnostic value of *LINC01234* in GC. A, ROC curve of *LINC01234* predicting STAD samples using FPKM value from TCGA. B, ROC curve of *LINC01234* predicting GC samples using △Ct value detected by qRT‐PCR

In the previous study, *LINC01234* was considered to be a potential diagnostic marker in GC based on the data of TCGA.^26^ Consequently, we evaluated the likely diagnostic value of *LINC01234* based on our own dataset. Initially, we performed a statistical analysis to examine the relationship between the clinicopathological factors and the expression level of *LINC01234*. As a result, we found differentiation of GC was associated with *LINC01234* expression, that means the lower the *LINC01234* expression is, the more the possibility for poor differentiation of GC tissues is (*P* < .05, Table 1). Besides, *P* value of the test for association between distal metastasis and *LINC01234* expression is <0.05. However, the sample size of GC patients with M1 stage is not enough (n = 5) that the result may be unbelievable. Other clinicopathological factors including age, gender, greatest tumor dimension, invasion depth, lymphatic metastasis, and TNM stage are not related to *LINC01234* level. In addition, we further explored the ability of differentiation of GC tissues from the normal adjacent tissues by a receiver operating characteristic (ROC) curve. The area under the ROC curve (AUC) was 0.888 for TCGA dataset (95% CI, 0.848‐0.929; *P* < .05, Figure 2A) while 0.664 for our qRT‐PCR results (95% CI, 0.581‐0.748; *P* < .05, Figure 2B), indicating that *LINC01234* plays a prominent role in GC tumorigenesis.

**Table 1:** Relationship between *LINC01234* expression level (ΔCt value) and clinicopathological factors of GC patients

Characteristics	Groups	Number of Patients (%)	Expression level (Mean ± SE)	*P*‐value
Age (y)				.079
	≧60	33 (39.76)	17.55 ± 2.14	
	<60	50 (60.24)	18.34 ± 1.88	
Gender				.619
	Male	63 (75.9)	17.96 ± 1.92	
	Female	20 (24.1)	18.22 ± 2.31	
Greatest tumor dimension (cm)				.474
	≧5	40 (48.19)	18.19 ± 1.91	
	<5	43 (51.81)	17.87 ± 2.12	
Invasion depth				.804
	T1/T2	14 (16.87)	18.15 ± 1.76	
	T3/T4	69 (88.13)	18 ± 2.07	
Differentiation				<.001*
	Well/Moderate	37 (44.58)	17.17 ± 1.65	
	Poor	46 (55.42)	18.71 ± 2.03	
Lymphatic metastasis				.377
	N0/N1	28 (33.73)	18.3 ± 1.93	
	N2/N3	55 (66.27)	17.88 ± 2.06	
Distal metastasis				.013^a^
	M0	78 (93.98)	18.16 ± 1.99	
	M1	5 (6.02)	15.88 ± 0.818	
TNM stage				.87
	I/II	22 (26.51)	18.09 ± 1.95	
	Ⅲ/Ⅳ	61 (73.49)	18 ± 2.05	

Abbreviation: SE, standard error.

a The sample size is so small that the result may be unbelievable even though *P* < .05.

* *P* < .05.

### 3.3 Potential functions of *LINC01234*

**Figure 3:**
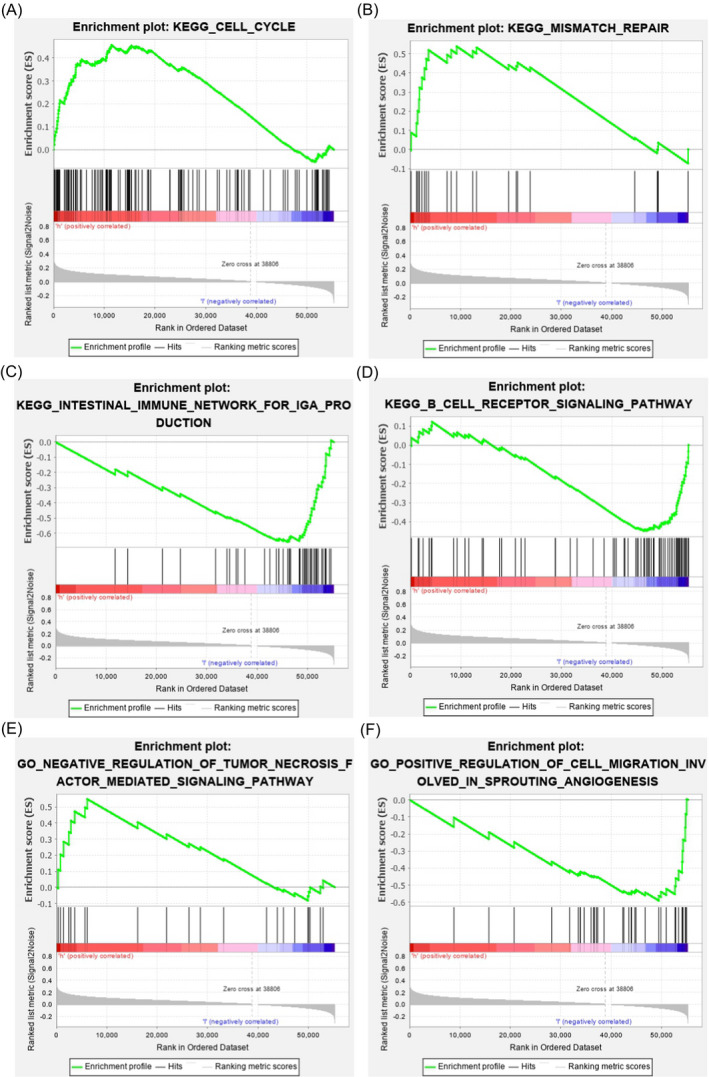
Potential functions of *LINC01234* in GC. GSEA showed that aberrant expression of *LINC01234* would affect the genes involved in cancer‐related pathways such as cell cycle (A), mismatch repair (B), intestinal immune network for IgA production (C), B‐cell receptor signaling pathway (D), negative regulation of tumor factor–mediated signaling pathway (E), and positive regulation of cell migration involved in sprouting angiogenesis (F)

To explore the potential functions of *LINC01234* in GC, we firstly divided the STAD patients from TCGA into two groups, low expression and high expression of *LINC01234* in cancer tissues, respectively. Secondly, GSEA was performed to investigate biological processes or pathways that were associated with *LINC01234.* Thus, the results showed *LINC01234* may be involved in cancer and immune‐related pathways such as cell cycle (Figure 3A), mismatch repair (Figure 3B), intestinal immune network for IgA production (Figure 3C), and B‐cell receptor signaling pathway (Figure 3D). In the case of GO BP, cancer‐associated functions were found such as negative regulation of tumor factor–mediated signaling pathway (Figure 3E) and positive regulation of cell migration are involved in sprouting angiogenesis (Figure 3F). These findings present a strong evidence that *LINC01234* has a major role in GC formation.

### 3.4 Regulatory network of *LINC01234*

**Figure 4:**
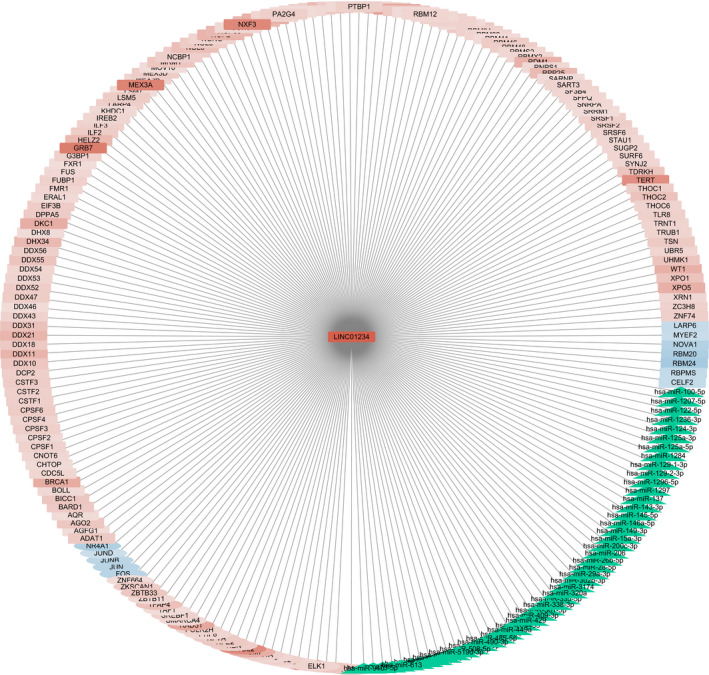
Regulatory network of *LINC01234* in GC. The central node is *LINC01234*. Rectangle nodes represent TF, circle nodes represent RBP, and green triangle nodes represent miRNA. Red color represents high expression in GC, while blue color represents low expression in GC. Color strength represents log2 value of fold change in GC tissues to adjacent normal tissues

LncRNAs have been discovered to interact with various types of molecules including DNA, miRNA, mRNA, and protein. To analyze the regulation mechanism of *LINC01234* in GC, we constructed a regulatory network of *LINC01234* by utilizing a series of bioinformatics tools and databases. This network included TF‐lncRNA regulation, miRNA‐lncRNA relationship, as well as lncRNA‐RBP interactions. In total, 31 TFs, 49 miRNAs, and 138 RBPs associated with *LINC01234* were achieved (Table S3, Figure 4).

#### 3.4.1 TF‐LINC01234 regulation

Some of the 31 TFs have thoroughly participated in GC development. For example, *FOXK2* inhibited the proliferation, invasion, and migration of GC cells, and its down‐regulation is related to poor prognosis in GC patients.^27^ Besides, *HDAC2* was significantly up‐regulated in various histopathologic grades of human GC, and the inactivation of *HDAC2* has been confirmed to reduce cell motility, cell invasion, clonal expansion, and tumor growth.^28^ Specifically, 2 TFs were found to co‐express with *LINC01234* according to the co‐expression network we previously constructed.^29^ They are *ELK1* and *ZNF664* (Table S4).

#### 3.4.2 miRNA‐LINC01234 regulation

A total of 49 miRNAs were predicted to regulate *LINC01234* in GC. Several of them have already been accepted to be correlated with GC progression. Also, the overexpression of miR‐1284 was reported to be a suppressor for GC by controlling over cell proliferation and apoptosis.^30^ In fact, a prior study showed miR‐1284 might modulate multidrug resistance of GC cells by targeting specific genes.^31^ The miR‐1297 expression found to be remarkably lower in GC tissue and suppress GC cell growth by inhibiting the expression of *CREB1*.^32^

#### 3.4.3 RBP‐LINC01234 regulation

A total of 138 RBPs were predicted to likely interact with *LINC01234*. Among them, a part of RBPs was already shown to be related to GC. For example, reports revealed *DDX21* could affect the proliferation of GC cells by up‐regulating levels of cyclin D1 and *CDK2*.^33^ Likewise, the suppression of *EIF3B* inhibits the proliferation and metastasis of GC by effectively modulating the expression of cancer‐related genes.^34^ Likewise, we also found 15 co‐expression relationships in RBP interactions such as *CPSF3*, *DDX18*, *DKC1*, and *FUS* (Table S4).

## 4 DISCUSSION

LncRNA is a type of non‐coding RNAs without the ability of encoding proteins; despite this, it has a regulating gene expression at chromatin modification, transcriptional, or post‐transcriptional levels.^35^ In addition, the polymorphisms in lncRNAs could be a risk of disease or cancer.^36^ In fact, an increasing number of lncRNAs have been identified to be related to numerous kinds of diseases,^37^ including GC. For example, through the expression analysis of metabolic pathway‐related lncRNAs and protein‐coding genes, a dozen of lncRNAs were functionally annotated and discovered to be important in GC.^38^ *DGCR9,* another lncRNA up‐regulated in GC, was shown to promote the tumorigenesis of GC*.* 39 Some of lncRNAs were even indeed considered as biomarkers for diagnosis or prognosis of GC*.* For instance, a metabolism‐related lncRNA, *RP11‐555H23.1*, was found to be a potential diagnostic biomarker in GC.^40^ Also, the expression level of *H19* in plasma could be served as a biomarker for patients with GC.^41^ Due to the regulatory role of lncRNAs, exploring new lncRNA biomarkers can help to explain the initiation and progression mechanism of GC. Nevertheless, still there are many unknown lncRNAs in GC nowadays.

The previous study found *LINC01234* is highly expressed in esophageal squamous cell carcinoma (ESCC). Also, it is one of the three lncRNAs that can be a signature to predict the survival time of ESCC patients accurately.^42^, ^43^, ^44^ Besides, it was likewise discovered to be up‐regulated in the GC in prior study.^26^ However, the functions and regulatory role of *LINC01234* need to be studied.

In this research, we identified that *LINC01234* was also highly expressed in GC tissues compared with adjacent non‐cancerous tissues. Later, we explored the associations between the expression level of *LINC01234* and clinical features through which we found *LINC01234* was correlated with differentiation of GC. LncRNAs usually interact with other kinds of molecules to involve in multiple biological processes. For example, the binding of lncRNA *OLC8* and *IL‐11* will impair the degradation of *IL‐1*1 mRNAs to accelerate GC development.^45^ Besides, combination of lncRNA and its target may increase the diagnostic value of lncRNA. Just as it was found by previous report that the combined use of *RP11‐19P22.6‐001* and its target *NOS2* may be useful to diagnose patients with GC.^46^ Thus, we further explored the potential functions and regulatory network of *LINC01234*. We identified 218 relationships of *LINC01234* in total. Among them, 17 associations including two pairs of TF regulation and 17 pairs of RBP interaction were found to be co‐expressed. One of 2 TFs, *ELK1*, is an important regulator and known to activate many lncRNAs including *TRPM2‐AS*, *MIR100HG*, and *HOXA10‐AS* in cancer until now,^47^, ^48^, ^49^ indicating *ELK1* may induce expression of *LINC01234* to promote tumor progression in GC too.

In conclusion, the results of this present study indicated that *LINC01234* expression is linked with the diagnostics of patients with GC and similarly may be involved in differentiation in GC through cell cycle or other cancer‐related pathways.
