# Supplementary material for: Function and regulation annotation of up‐regulated long non‐coding RNA LINC01234 in gastric cancer
Source: J Clin Lab Anal. 2020 Feb 3;34(5):e23210. doi: 10.1002/jcla.23210 (PMC7246363; doi:10.1002/jcla.23210)
Supplement: Supplementary file 1 [file JCLA-34-e23210-s001.docx]

**Supplementary Table 1**

TCGA-BR-8369-01

TCGA-HU-A4G9-01

TCGA-HU-A4GP-11

TCGA-CG-5732-01

TCGA-CD-5798-01

TCGA-BR-6709-01

TCGA-VQ-A8PD-01

TCGA-B7-5818-01

TCGA-CG-4462-01

TCGA-F1-6177-01

TCGA-VQ-A925-01

TCGA-VQ-A94P-01

TCGA-BR-6852-01

TCGA-IN-A7NR-01

TCGA-BR-8679-01

TCGA-BR-6565-01

TCGA-CG-4460-01

TCGA-VQ-A91X-01

TCGA-VQ-A8DZ-01

TCGA-VQ-A8E2-01

TCGA-IN-8663-01

TCGA-IN-8462-01

TCGA-RD-A7BS-01

TCGA-IN-8663-11

TCGA-CG-5722-11

TCGA-BR-4191-01

TCGA-D7-6522-01

TCGA-BR-6563-01

TCGA-BR-8683-01

TCGA-R5-A7ZI-01

TCGA-BR-8686-01

TCGA-BR-4367-01

TCGA-CD-A486-01

TCGA-VQ-A8P3-01

TCGA-BR-4256-01

TCGA-CG-5728-11

TCGA-BR-6457-11

TCGA-BR-8372-01

TCGA-BR-8366-01

TCGA-BR-8291-01

TCGA-IN-7808-01

TCGA-BR-8297-01

TCGA-VQ-A8PK-01

TCGA-D7-6822-01

TCGA-BR-8058-01

TCGA-IN-A6RS-01

TCGA-HU-8238-01

TCGA-BR-7717-01

TCGA-VQ-A8PP-01

TCGA-HJ-7597-01

TCGA-VQ-A8P8-01

TCGA-CD-A487-01

TCGA-FP-A4BF-01

TCGA-BR-6453-11

TCGA-D7-A6EX-01

TCGA-HU-A4H4-01

TCGA-VQ-A8DT-01

TCGA-HU-A4GF-01

TCGA-BR-8367-01

TCGA-BR-7901-01

TCGA-BR-6707-01

TCGA-HU-A4H5-01

TCGA-MX-A666-01

TCGA-BR-8682-01

TCGA-CG-4436-01

TCGA-D7-A6F2-01

TCGA-HU-A4H8-01

TCGA-BR-6455-01

TCGA-VQ-A8P5-01

TCGA-BR-8486-01

TCGA-CG-4444-01

TCGA-BR-A4CS-01

TCGA-FP-A8CX-01

TCGA-VQ-AA6D-01

TCGA-VQ-A91U-01

TCGA-ZQ-A9CR-01

TCGA-D7-A4Z0-01

TCGA-BR-A4J5-01

TCGA-BR-4361-01

TCGA-BR-6454-11

TCGA-BR-7196-01

TCGA-CG-4469-01

TCGA-BR-6452-01

TCGA-HU-A4GD-01

TCGA-CG-5730-11

TCGA-RD-A8N2-01

TCGA-RD-A8N6-01

TCGA-BR-7715-01

TCGA-FP-7829-11

TCGA-HU-8238-11

TCGA-D7-6521-01

TCGA-D7-A6EV-01

TCGA-BR-4187-01

TCGA-BR-A4J9-01

TCGA-VQ-AA64-01

TCGA-VQ-A922-01

TCGA-BR-8678-01

TCGA-IN-AB1V-11

TCGA-CG-5721-01

TCGA-BR-A4CR-01

TCGA-BR-6458-01

TCGA-IN-AB1X-11

TCGA-BR-8484-01

TCGA-BR-6456-01

TCGA-IP-7968-11

TCGA-D7-A6EY-01

TCGA-BR-8364-01

TCGA-ZA-A8F6-01

TCGA-HU-A4GC-01

TCGA-HU-A4GY-01

TCGA-CD-5813-01

TCGA-CD-8534-01

TCGA-VQ-AA6F-01

TCGA-BR-8081-01

TCGA-HU-A4GT-01

TCGA-BR-7723-01

TCGA-BR-8289-01

TCGA-IP-7968-01

TCGA-CG-4440-01

TCGA-F1-6875-01

TCGA-HU-8244-01

TCGA-R5-A805-01

TCGA-FP-8209-01

TCGA-3M-AB47-01

TCGA-KB-A93J-01

TCGA-IN-A6RN-01

TCGA-BR-7703-11

TCGA-D7-6526-01

TCGA-CG-5733-11

TCGA-VQ-A924-01

TCGA-CG-4443-01

TCGA-D7-A4YU-01

TCGA-IN-A6RJ-01

TCGA-IN-A6RO-01

TCGA-VQ-A928-01

TCGA-CG-4304-01

TCGA-HU-A4GP-01

TCGA-CG-4305-01

TCGA-R5-A7ZE-01

TCGA-BR-6453-01

TCGA-HF-7134-01

TCGA-HU-A4GJ-01

TCGA-VQ-AA6A-01

TCGA-D7-A6EZ-01

TCGA-D7-6528-01

TCGA-BR-4267-01

TCGA-VQ-A91A-01

TCGA-VQ-A8PE-01

TCGA-BR-7722-01

TCGA-BR-A4PF-01

TCGA-HU-A4HB-11

TCGA-CG-5722-01

TCGA-CG-4306-01

TCGA-BR-A4J7-01

TCGA-CG-5734-01

TCGA-BR-4279-01

TCGA-HU-A4G2-01

TCGA-BR-8059-01

TCGA-CD-8535-01

TCGA-BR-7851-01

TCGA-D7-8579-01

TCGA-IN-A7NU-01

TCGA-HU-A4GN-11

TCGA-BR-8588-01

TCGA-VQ-A8PJ-01

TCGA-IN-AB1X-01

TCGA-CG-5724-01

TCGA-BR-7851-11

TCGA-VQ-A8E0-01

TCGA-D7-6524-01

TCGA-D7-A748-01

TCGA-VQ-A91K-01

TCGA-BR-4370-01

TCGA-CG-4438-01

TCGA-VQ-A8PC-01

TCGA-CD-A48C-01

TCGA-BR-8382-01

TCGA-R5-A7ZF-01

TCGA-IN-AB1V-01

TCGA-KB-A93G-01

TCGA-FP-7916-01

TCGA-D7-8572-01

TCGA-R5-A7ZR-01

TCGA-CD-5799-01

TCGA-VQ-A8PU-01

TCGA-VQ-A91D-01

TCGA-BR-7715-11

TCGA-BR-4363-01

TCGA-BR-7707-01

TCGA-VQ-A94T-01

TCGA-BR-A44T-01

TCGA-HU-A4HD-01

TCGA-BR-6802-11

TCGA-BR-8368-01

TCGA-BR-8080-01

TCGA-BR-6564-01

TCGA-BR-A4J4-01

TCGA-BR-8384-01

TCGA-BR-7716-01

TCGA-FP-7829-01

TCGA-VQ-A8E7-01

TCGA-VQ-AA6K-01

TCGA-VQ-A92D-01

TCGA-RD-A8N0-01

TCGA-BR-8380-01

TCGA-BR-8592-01

TCGA-CG-5718-01

TCGA-F1-6874-01

TCGA-RD-A8N9-01

TCGA-CG-4301-01

TCGA-BR-6454-01

TCGA-IN-A6RL-01

TCGA-BR-6457-01

TCGA-MX-A5UG-01

TCGA-CG-4475-01

TCGA-IN-A6RI-01

TCGA-B7-A5TI-01

TCGA-BR-A4J6-01

TCGA-D7-A74A-01

TCGA-CG-4441-01

TCGA-BR-8591-01

TCGA-IN-7806-11

TCGA-CD-5804-01

TCGA-BR-6705-01

TCGA-MX-A5UJ-01

TCGA-CD-8526-01

TCGA-BR-8361-01

TCGA-BR-A44U-01

TCGA-BR-4366-01

TCGA-VQ-A91E-01

TCGA-VQ-A8PH-01

TCGA-D7-A6F0-01

TCGA-VQ-A8PO-01

TCGA-VQ-A8PF-01

TCGA-CG-5734-11

TCGA-RD-A7BT-01

TCGA-BR-8295-01

TCGA-CG-4437-01

TCGA-HU-A4H3-01

TCGA-HU-A4GC-11

TCGA-BR-6803-01

TCGA-VQ-A94U-01

TCGA-BR-6802-01

TCGA-HF-A5NB-01

TCGA-BR-8373-01

TCGA-D7-8570-01

TCGA-FP-8631-01

TCGA-RD-A8MV-01

TCGA-CD-8531-01

TCGA-VQ-A8PX-01

TCGA-HU-A4G3-01

TCGA-BR-7959-01

TCGA-VQ-A94R-01

TCGA-BR-4368-01

TCGA-FP-7735-11

TCGA-HU-A4GY-11

TCGA-RD-A8N1-01

TCGA-3M-AB46-01

TCGA-BR-A4J8-01

TCGA-F1-A448-01

TCGA-BR-8487-01

TCGA-HU-8602-01

TCGA-VQ-A91Z-01

TCGA-BR-8690-01

TCGA-RD-A8NB-01

TCGA-CD-8528-01

TCGA-D7-8573-01

TCGA-CG-5716-01

TCGA-BR-4294-01

TCGA-CD-8532-01

TCGA-HU-A4GU-01

TCGA-HU-A4H0-01

TCGA-BR-8077-01

TCGA-VQ-A91Q-01

TCGA-BR-7716-11

TCGA-HU-8610-01

TCGA-HU-8249-01

TCGA-KB-A93H-01

TCGA-SW-A7EA-01

TCGA-CD-5803-01

TCGA-D7-6520-01

TCGA-D7-6818-01

TCGA-CD-8525-01

TCGA-BR-7704-11

TCGA-CD-8533-01

TCGA-FP-8210-01

TCGA-D7-6519-01

TCGA-HU-8608-01

TCGA-CG-4466-01

TCGA-CG-4442-01

TCGA-VQ-A94O-01

TCGA-CG-5720-11

TCGA-RD-A7BW-01

TCGA-F1-A72C-01

TCGA-BR-7704-01

TCGA-VQ-A8PQ-01

TCGA-RD-A8MW-01

TCGA-RD-A8N4-01

TCGA-HU-A4G8-01

TCGA-BR-7717-11

TCGA-BR-4253-01

TCGA-BR-A4IV-01

TCGA-HU-A4GH-01

TCGA-BR-8381-01

TCGA-D7-A747-01

TCGA-BR-4357-01

TCGA-BR-8060-11

TCGA-HU-A4H6-01

TCGA-KB-A6F7-01

TCGA-D7-5577-01

TCGA-VQ-A8DV-01

TCGA-CD-8530-01

TCGA-VQ-A927-01

TCGA-D7-6525-01

TCGA-FP-8211-01

TCGA-BR-8676-01

TCGA-IN-7806-01

TCGA-HF-7133-01

TCGA-VQ-A923-01

TCGA-CG-5717-01

TCGA-CD-5801-01

TCGA-BR-4371-01

TCGA-VQ-A91N-01

TCGA-BR-8589-01

TCGA-CG-5719-01

TCGA-D7-8574-01

TCGA-B7-A5TN-01

TCGA-BR-7957-01

TCGA-CG-5720-01

TCGA-CD-5800-01

TCGA-BR-4201-01

TCGA-HF-7131-01

TCGA-CD-8529-01

TCGA-IN-A7NT-01

TCGA-VQ-AA6J-01

TCGA-BR-8371-01

TCGA-CG-4476-01

TCGA-BR-4280-01

TCGA-BR-6566-01

TCGA-D7-A4YX-01

TCGA-BR-8485-01

TCGA-B7-A5TK-01

TCGA-BR-7197-01

TCGA-CD-8524-01

TCGA-D7-6527-01

TCGA-VQ-AA68-01

TCGA-MX-A663-01

TCGA-VQ-A8P2-01

TCGA-VQ-A8PM-01

TCGA-VQ-A8DU-01

TCGA-CG-5723-01

TCGA-BR-4257-01

TCGA-HU-A4H2-01

TCGA-BR-8483-01

TCGA-BR-7958-01

TCGA-CG-5721-11

TCGA-BR-8296-01

TCGA-FP-A9TM-01

TCGA-CG-4477-01

TCGA-HU-A4GQ-01

TCGA-BR-8284-01

TCGA-BR-8590-01

TCGA-CD-8527-01

TCGA-CG-4465-01

TCGA-BR-8677-01

TCGA-R5-A7O7-01

TCGA-VQ-AA69-01

TCGA-BR-8286-01

TCGA-D7-5578-01

TCGA-BR-6710-01

TCGA-IN-A6RR-01

TCGA-VQ-A8PB-01

TCGA-D7-6815-01

TCGA-HU-A4HB-01

TCGA-BR-8365-01

TCGA-VQ-A91Y-01

TCGA-BR-4369-01

TCGA-FP-7998-01

TCGA-BR-8687-01

TCGA-VQ-AA6G-01

TCGA-CD-A4MG-01

TCGA-HU-8604-01

TCGA-BR-A4QL-01

TCGA-RD-A8N5-01

TCGA-BR-6801-01

TCGA-HU-A4GH-11

TCGA-CD-A4MH-01

TCGA-HU-A4GX-01

TCGA-EQ-8122-01

TCGA-RD-A7C1-01

TCGA-SW-A7EB-01

TCGA-HF-7132-01

TCGA-FP-8099-01

TCGA-CG-5726-01

TCGA-D7-8578-01

TCGA-B7-A5TJ-01

TCGA-CD-A489-01

TCGA-BR-8680-01

TCGA-D7-8576-01

TCGA-BR-8060-01

TCGA-VQ-A91V-01

TCGA-VQ-A91S-01

TCGA-CG-5725-01

TCGA-D7-8575-01

TCGA-CD-A48A-01

TCGA-FP-7735-01

TCGA-VQ-A8E3-01
