# Supplementary material for: Function and regulation annotation of up‐regulated long non‐coding RNA LINC01234 in gastric cancer
Source: J Clin Lab Anal. 2020 Feb 3;34(5):e23210. doi: 10.1002/jcla.23210 (PMC7246363; doi:10.1002/jcla.23210)
Supplement: Supplementary file 2 [file JCLA-34-e23210-s002.docx]

**Supplementary Table 2**

| **lncRNA** | **p-value** | **FDR** | **cancer_mean** | **normal_mean** | **FC** |
| --- | --- | --- | --- | --- | --- |
| AADACL2-AS1 | 3.30E-08 | 2.16E-07 | 0.219389 | 0.059259 | 2.005471 |
| AB019440.50 | 0.000401 | 0.001089 | 0.323665 | 0.033487 | 3.173838 |
| ABALON | 3.38E-23 | 2.30E-21 | 1.052459 | 0.330898 | 2.674549 |
| AC000111.6 | 0.000188 | 0.000557 | 0.855625 | 0.061261 | 5.925964 |
| AC002076.10 | 5.64E-14 | 9.73E-13 | 0.171962 | 0.01957 | 2.2745 |
| AC002116.7 | 1.90E-17 | 5.63E-16 | 0.888098 | 0.318396 | 2.361632 |
| AC002128.5 | 8.81E-19 | 3.22E-17 | 0.855646 | 0.24576 | 2.763896 |
| AC002331.1 | 6.51E-16 | 1.53E-14 | 1.054869 | 0.223172 | 3.573542 |
| AC002398.13 | 2.59E-21 | 1.33E-19 | 1.348584 | 0.584019 | 2.117754 |
| AC004009.3 | 3.25E-12 | 4.21E-11 | 0.788481 | 0.173577 | 3.247647 |
| AC004231.2 | 0.002832 | 0.006052 | 0.351424 | 0.042438 | 3.169261 |
| AC004593.3 | 3.38E-07 | 1.82E-06 | 0.304716 | 0.100603 | 2.017495 |
| AC004837.5 | 2.24E-08 | 1.51E-07 | 0.368326 | 0.123246 | 2.097806 |
| AC004870.4 | 1.06E-10 | 1.08E-09 | 0.176464 | 0.016919 | 2.364572 |
| AC004988.1 | 3.62E-47 | 6.61E-44 | 0.180588 | 0.016475 | 2.409 |
| AC005104.3 | 4.22E-10 | 3.85E-09 | 0.813293 | 0.351827 | 2.021332 |
| AC005256.1 | 0.000156 | 0.000473 | 1.355722 | 0.450921 | 2.642344 |
| AC005307.4 | 0.014816 | 0.025604 | 0.138031 | 0.009426 | 2.17528 |
| AC005522.7 | 4.41E-15 | 8.86E-14 | 0.335816 | 0.090559 | 2.287037 |
| AC005534.8 | 3.84E-34 | 9.81E-32 | 0.268409 | 0.038775 | 2.654722 |
| AC005546.2 | 3.45E-09 | 2.72E-08 | 0.619868 | 0.247742 | 2.07012 |
| AC005550.3 | 0.005204 | 0.010291 | 1.903823 | 0.797911 | 2.231651 |
| AC006000.5 | 6.10E-21 | 2.98E-19 | 0.132654 | 0.002354 | 2.273045 |
| AC006042.6 | 1.23E-30 | 2.18E-28 | 2.551237 | 0.639329 | 3.586004 |
| AC006262.5 | 0.019324 | 0.03221 | 2.055172 | 0.770851 | 2.474787 |
| AC006273.4 | 6.93E-19 | 2.57E-17 | 0.225946 | 0.041749 | 2.299468 |
| AC006273.5 | 1.40E-28 | 1.97E-26 | 1.113466 | 0.232137 | 3.653511 |
| AC006277.2 | 7.49E-13 | 1.09E-11 | 0.630908 | 0.151967 | 2.900816 |
| AC007078.4 | 2.33E-12 | 3.10E-11 | 0.268669 | 0.06789 | 2.195898 |
| AC007099.1 | 2.38E-06 | 1.08E-05 | 0.361077 | 0.00496 | 4.392894 |
| AC007128.1 | 2.96E-45 | 4.20E-42 | 0.568381 | 0.011136 | 6.014081 |
| AC007249.3 | 7.33E-06 | 2.99E-05 | 0.449147 | 0.144186 | 2.248889 |
| AC007277.3 | 0.009837 | 0.017918 | 0.238979 | 0.041862 | 2.389499 |
| AC007405.8 | 1.61E-15 | 3.55E-14 | 0.141958 | 0.002359 | 2.363819 |
| AC007566.10 | 0.000102 | 0.000322 | 1.308155 | 0.589987 | 2.040842 |
| AC007773.2 | 6.52E-16 | 1.53E-14 | 0.448213 | 0.171954 | 2.015832 |
| AC007950.1 | 0.019857 | 0.03297 | 0.209258 | 0.032012 | 2.342644 |
| AC008440.10 | 2.38E-12 | 3.17E-11 | 0.935925 | 0.124583 | 4.612651 |
| AC008746.12 | 8.34E-21 | 4.04E-19 | 0.779436 | 0.263683 | 2.418139 |
| AC009005.2 | 2.36E-18 | 8.12E-17 | 2.385795 | 0.759585 | 2.891857 |
| AC009095.4 | 7.08E-16 | 1.65E-14 | 0.52619 | 0.21016 | 2.018925 |
| AC009120.5 | 2.05E-10 | 1.97E-09 | 1.355641 | 0.593487 | 2.099016 |
| AC009410.1 | 3.17E-10 | 2.97E-09 | 0.273705 | 0.008259 | 3.451952 |
| AC010524.2 | 1.61E-10 | 1.58E-09 | 0.362207 | 0.090119 | 2.431144 |
| AC010761.13 | 3.53E-15 | 7.22E-14 | 0.93755 | 0.342084 | 2.346952 |
| AC010967.2 | 5.92E-14 | 1.02E-12 | 0.145516 | 0.016466 | 2.108048 |
| AC011288.2 | 8.97E-26 | 9.25E-24 | 0.504062 | 0.023544 | 4.889462 |
| AC011298.2 | 1.04E-05 | 4.11E-05 | 0.327922 | 0.034715 | 3.1765 |
| AC011513.4 | 5.45E-12 | 6.81E-11 | 0.303578 | 0.050982 | 2.673018 |
| AC011523.2 | 0.008239 | 0.015378 | 0.385372 | 0.072191 | 2.818799 |
| AC011747.3 | 2.14E-26 | 2.40E-24 | 0.188901 | 0.032389 | 2.182216 |
| AC011747.4 | 4.47E-30 | 7.62E-28 | 0.277921 | 0.066782 | 2.265962 |
| AC011747.6 | 1.71E-08 | 1.19E-07 | 0.146484 | 0.009256 | 2.256029 |
| AC011997.1 | 1.62E-14 | 2.99E-13 | 0.212351 | 0.052252 | 2.05154 |
| AC012363.4 | 1.05E-52 | 4.47E-49 | 0.740082 | 0.022251 | 6.871796 |
| AC012499.1 | 3.04E-05 | 0.000108 | 0.11295 | 0.004904 | 2.029942 |
| AC012531.25 | 7.61E-37 | 3.36E-34 | 0.306593 | 0.010068 | 3.694018 |
| AC013275.2 | 4.31E-06 | 1.85E-05 | 0.453094 | 0.103131 | 2.722846 |
| AC015849.16 | 1.65E-38 | 8.81E-36 | 0.194468 | 0.02705 | 2.317728 |
| AC015849.19 | 1.27E-19 | 5.27E-18 | 2.901968 | 1.135437 | 2.429884 |
| AC016292.1 | 8.51E-08 | 5.14E-07 | 0.34502 | 0.101593 | 2.20752 |
| AC016735.1 | 2.56E-12 | 3.39E-11 | 4.673719 | 1.410485 | 3.160389 |
| AC016735.2 | 3.21E-06 | 1.41E-05 | 2.624755 | 0.34921 | 6.06566 |
| AC017002.1 | 1.08E-08 | 7.79E-08 | 0.304089 | 0.093826 | 2.084795 |
| AC017060.1 | 3.73E-12 | 4.82E-11 | 1.483398 | 0.232547 | 4.761427 |
| AC017074.2 | 6.43E-09 | 4.83E-08 | 0.874957 | 0.336481 | 2.233675 |
| AC018890.6 | 1.66E-07 | 9.48E-07 | 0.178614 | 0.028773 | 2.163606 |
| AC024560.2 | 0.000162 | 0.000489 | 0.176366 | 0.028396 | 2.152445 |
| AC025016.1 | 4.04E-05 | 0.000139 | 0.121784 | 0.00496 | 2.113025 |
| AC026471.6 | 5.17E-17 | 1.42E-15 | 0.748156 | 0.189481 | 2.929916 |
| AC027119.1 | 6.34E-10 | 5.62E-09 | 0.220884 | 0.009024 | 2.94325 |
| AC046143.3 | 8.23E-17 | 2.21E-15 | 0.893558 | 0.376987 | 2.082988 |
| AC064834.1 | 0.029278 | 0.046365 | 0.216725 | 0.007024 | 2.959377 |
| AC068134.6 | 4.39E-11 | 4.80E-10 | 0.110693 | 0.004418 | 2.017792 |
| AC068580.7 | 2.01E-10 | 1.95E-09 | 1.357924 | 0.043162 | 10.18375 |
| AC069277.2 | 1.37E-13 | 2.21E-12 | 0.139488 | 0.019527 | 2.003635 |
| AC073283.7 | 2.42E-39 | 1.47E-36 | 0.546157 | 0.080608 | 3.57768 |
| AC073333.8 | 1.52E-29 | 2.40E-27 | 0.63268 | 0.169446 | 2.719208 |
| AC074117.10 | 6.11E-48 | 1.30E-44 | 2.172688 | 0.788084 | 2.559093 |
| AC074389.5 | 0.001371 | 0.003212 | 0.463815 | 0.010096 | 5.121116 |
| AC079354.5 | 9.01E-11 | 9.39E-10 | 0.204657 | 0.046963 | 2.073012 |
| AC079466.1 | 0.026372 | 0.042309 | 0.423509 | 0.003185 | 5.073484 |
| AC083843.1 | 2.77E-19 | 1.10E-17 | 1.086508 | 0.417623 | 2.292225 |
| AC083884.8 | 7.72E-15 | 1.50E-13 | 0.433889 | 0.157993 | 2.069392 |
| AC087491.2 | 0.000112 | 0.00035 | 1.252885 | 0.309714 | 3.302022 |
| AC091729.8 | 3.99E-20 | 1.76E-18 | 0.176352 | 0.027282 | 2.17117 |
| AC092198.1 | 1.50E-11 | 1.76E-10 | 0.195104 | 0.010914 | 2.660649 |
| AC092415.1 | 2.11E-18 | 7.32E-17 | 0.213186 | 0.01056 | 2.832729 |
| AC093375.1 | 4.23E-20 | 1.85E-18 | 0.535032 | 0.14004 | 2.645528 |
| AC093620.5 | 8.35E-15 | 1.60E-13 | 0.927321 | 0.413176 | 2.00189 |
| AC093732.1 | 1.96E-16 | 5.00E-15 | 0.591406 | 0.110819 | 3.279624 |
| AC098973.2 | 1.36E-15 | 3.03E-14 | 1.007454 | 0.030251 | 8.502438 |
| AC104088.1 | 2.59E-15 | 5.51E-14 | 0.718447 | 0.211383 | 2.628423 |
| AC104534.2 | 7.85E-06 | 3.19E-05 | 6.617405 | 1.62314 | 3.89835 |
| AC104654.1 | 9.86E-11 | 1.02E-09 | 0.346188 | 0.016652 | 3.82494 |
| AC104654.2 | 1.96E-17 | 5.78E-16 | 0.417481 | 0.024856 | 4.144612 |
| AC104809.4 | 2.63E-05 | 9.40E-05 | 0.335425 | 0.025522 | 3.468912 |
| AC107081.5 | 8.52E-15 | 1.63E-13 | 0.499532 | 0.174566 | 2.183565 |
| AC108463.1 | 4.66E-32 | 9.02E-30 | 0.36092 | 0.057248 | 2.931164 |
| AC112721.2 | 5.02E-11 | 5.44E-10 | 0.242747 | 0.058286 | 2.165357 |
| AC114730.3 | 4.38E-23 | 2.93E-21 | 0.447418 | 0.037174 | 3.990673 |
| AC114730.5 | 2.10E-20 | 9.65E-19 | 0.176166 | 0.01281 | 2.448075 |
| AC129492.6 | 6.04E-15 | 1.19E-13 | 0.418684 | 0.103199 | 2.552591 |
| AC141928.1 | 0.00241 | 0.005274 | 0.380684 | 0.090268 | 2.526351 |
| AC226118.1 | 1.78E-14 | 3.27E-13 | 0.370188 | 0.074145 | 2.699982 |
| ADARB2-AS1 | 0.02454 | 0.039729 | 0.194299 | 0.031577 | 2.236703 |
| AF011889.2 | 2.07E-10 | 1.99E-09 | 0.420512 | 0.156452 | 2.029663 |
| AF064858.11 | 0.000297 | 0.000833 | 0.583361 | 0.185249 | 2.395667 |
| AF124730.4 | 2.99E-14 | 5.32E-13 | 0.416024 | 0.055333 | 3.322043 |
| AF127577.8 | 9.29E-19 | 3.38E-17 | 0.432584 | 0.009947 | 4.844011 |
| AFAP1-AS1 | 6.24E-17 | 1.69E-15 | 6.938009 | 1.34948 | 4.855542 |
| AGAP2-AS1 | 2.08E-16 | 5.29E-15 | 5.902785 | 2.826371 | 2.051273 |
| AL121578.2 | 7.06E-12 | 8.68E-11 | 0.11828 | 0.00528 | 2.073325 |
| AL133493.2 | 3.02E-08 | 1.99E-07 | 0.365573 | 0.047605 | 3.154182 |
| AL162759.1 | 4.03E-06 | 1.73E-05 | 0.203459 | 0.016497 | 2.604869 |
| AL163953.2 | 6.39E-12 | 7.92E-11 | 0.432177 | 0.092941 | 2.758234 |
| AL450992.2 | 1.70E-14 | 3.15E-13 | 4.151318 | 1.429397 | 2.779735 |
| AL773572.7 | 0.008856 | 0.016345 | 0.471445 | 0.088379 | 3.033484 |
| ALG13-AS1 | 1.42E-07 | 8.22E-07 | 1.479413 | 0.613347 | 2.214089 |
| ANO1-AS2 | 3.33E-13 | 5.09E-12 | 0.170754 | 0.028148 | 2.112826 |
| AP000344.3 | 4.52E-09 | 3.50E-08 | 0.268578 | 0.054433 | 2.386648 |
| AP000487.6 | 2.86E-15 | 6.03E-14 | 0.639583 | 0.207662 | 2.403878 |
| AP000593.7 | 7.37E-07 | 3.72E-06 | 0.269661 | 0.083806 | 2.01115 |
| AP000695.4 | 1.70E-19 | 6.95E-18 | 0.802894 | 0.217796 | 2.841114 |
| AP000695.6 | 2.57E-26 | 2.78E-24 | 0.715661 | 0.132653 | 3.50591 |
| AP001065.15 | 3.35E-16 | 8.26E-15 | 3.142334 | 0.651803 | 4.312747 |
| AP001601.2 | 0.000135 | 0.000415 | 0.137692 | 0.009112 | 2.178425 |
| AP001625.6 | 7.04E-07 | 3.57E-06 | 1.249109 | 0.566432 | 2.024378 |
| AP001626.1 | 0.001329 | 0.003125 | 0.608008 | 0.104907 | 3.45527 |
| AP001628.6 | 1.76E-13 | 2.81E-12 | 0.828996 | 0.202112 | 3.075008 |
| AP002954.4 | 1.46E-09 | 1.22E-08 | 0.54336 | 0.185374 | 2.254445 |
| AP003900.6 | 2.09E-07 | 1.17E-06 | 0.208142 | 0.009854 | 2.805028 |
| AP006748.1 | 8.05E-14 | 1.36E-12 | 0.252358 | 0.03196 | 2.670198 |
| ARMC2-AS1 | 5.30E-07 | 2.76E-06 | 0.265358 | 0.0775 | 2.058349 |
| ASMTL-AS1 | 4.24E-19 | 1.63E-17 | 1.63359 | 0.632683 | 2.366083 |
| BANCR | 0.005925 | 0.0115 | 2.724616 | 0.030913 | 21.5763 |
| BBOX1-AS1 | 5.12E-11 | 5.54E-10 | 2.637412 | 0.894264 | 2.753204 |
| BCAR4 | 0.001414 | 0.003301 | 0.609017 | 0.013447 | 6.249786 |
| BLACAT1 | 1.96E-34 | 5.34E-32 | 1.731643 | 0.164147 | 6.934186 |
| BOK-AS1 | 1.67E-05 | 6.27E-05 | 0.357646 | 0.032577 | 3.451922 |
| bP-2171C21.3 | 0.00015 | 0.000459 | 0.2671 | 0.041171 | 2.600395 |
| BX470102.3 | 1.45E-20 | 6.80E-19 | 2.87402 | 0.793087 | 3.330044 |
| C10orf55 | 7.47E-13 | 1.09E-11 | 0.347174 | 0.123293 | 2.002634 |
| C10orf91 | 6.54E-08 | 4.03E-07 | 0.593367 | 0.141915 | 2.866156 |
| C17orf77 | 2.56E-05 | 9.21E-05 | 0.169515 | 0.030359 | 2.067476 |
| C1orf195 | 2.20E-17 | 6.46E-16 | 0.812551 | 0.29875 | 2.288529 |
| C1QTNF1-AS1 | 2.34E-14 | 4.23E-13 | 0.271062 | 0.068386 | 2.203637 |
| C1RL-AS1 | 1.64E-22 | 1.02E-20 | 0.992085 | 0.41433 | 2.123314 |
| C2-AS1 | 2.56E-18 | 8.71E-17 | 0.188391 | 0.034442 | 2.145104 |
| C2orf48 | 2.35E-26 | 2.59E-24 | 0.583461 | 0.083014 | 3.73447 |
| C6orf99 | 5.60E-13 | 8.35E-12 | 0.677302 | 0.269282 | 2.1049 |
| C8orf31 | 1.60E-14 | 2.96E-13 | 1.876193 | 0.211763 | 6.338765 |
| CAPN10-AS1 | 1.28E-19 | 5.28E-18 | 0.478782 | 0.17521 | 2.103058 |
| CASC19 | 2.56E-14 | 4.60E-13 | 3.270245 | 0.489035 | 5.72164 |
| CASC20 | 6.67E-07 | 3.39E-06 | 0.191251 | 0.031111 | 2.221404 |
| CASC21 | 5.07E-07 | 2.65E-06 | 1.041365 | 0.07331 | 6.585699 |
| CASC8 | 1.25E-11 | 1.48E-10 | 0.923515 | 0.217286 | 3.225845 |
| CASC9 | 7.11E-33 | 1.65E-30 | 3.558071 | 0.387864 | 7.498134 |
| CASK-AS1 | 7.64E-22 | 4.36E-20 | 0.180071 | 0.033288 | 2.101251 |
| CATIP-AS2 | 1.94E-11 | 2.24E-10 | 0.491661 | 0.143367 | 2.431146 |
| CCAT1 | 1.48E-15 | 3.28E-14 | 4.037739 | 0.958746 | 3.90815 |
| CCDC144NL-AS1 | 5.34E-10 | 4.79E-09 | 0.50289 | 0.06915 | 3.56423 |
| CELSR3-AS1 | 3.34E-16 | 8.25E-15 | 1.348674 | 0.516323 | 2.350509 |
| CRAT40 | 1.69E-05 | 6.34E-05 | 0.246763 | 0.012619 | 3.079091 |
| CTA-126B4.7 | 9.86E-24 | 7.37E-22 | 0.756635 | 0.13697 | 3.614948 |
| CTA-217C2.2 | 7.82E-11 | 8.29E-10 | 1.101958 | 0.403918 | 2.385226 |
| CTA-228A9.3 | 6.16E-16 | 1.45E-14 | 1.473343 | 0.352662 | 3.475759 |
| CTA-305I2.1 | 2.98E-13 | 4.60E-12 | 0.781804 | 0.324353 | 2.077996 |
| CTA-363E6.5 | 3.06E-22 | 1.85E-20 | 0.291018 | 0.057429 | 2.48377 |
| CTA-363E6.6 | 2.37E-15 | 5.08E-14 | 2.424363 | 0.704863 | 3.136389 |
| CTA-384D8.31 | 0.000134 | 0.000412 | 4.016038 | 0.270289 | 11.11574 |
| CTA-384D8.35 | 0.000201 | 0.000593 | 1.549619 | 0.604807 | 2.340527 |
| CTA-384D8.36 | 2.84E-19 | 1.12E-17 | 3.033669 | 1.318371 | 2.209344 |
| CTA-398F10.2 | 2.04E-07 | 1.15E-06 | 0.513986 | 0.192364 | 2.100072 |
| CTA-989H11.1 | 1.67E-13 | 2.67E-12 | 1.055259 | 0.436177 | 2.154622 |
| CTB-113D17.1 | 3.16E-13 | 4.85E-12 | 0.273366 | 0.074181 | 2.143547 |
| CTB-119C2.1 | 7.24E-17 | 1.96E-15 | 0.605068 | 0.233157 | 2.11632 |
| CTB-131K11.1 | 2.95E-24 | 2.30E-22 | 11.17415 | 5.493123 | 2.015716 |
| CTB-13F3.1 | 8.38E-14 | 1.41E-12 | 1.401297 | 0.487319 | 2.556186 |
| CTB-186G2.4 | 6.57E-09 | 4.92E-08 | 1.326008 | 0.608151 | 2.013705 |
| CTB-1I21.1 | 0.000897 | 0.002207 | 0.227219 | 0 | 3.272194 |
| CTB-58E17.5 | 5.32E-17 | 1.45E-15 | 0.421933 | 0.152951 | 2.063373 |
| CTB-58E17.9 | 3.10E-18 | 1.04E-16 | 0.280578 | 0.080464 | 2.108891 |
| CTC-1337H24.4 | 0.000174 | 0.000521 | 0.367314 | 0.112713 | 2.196925 |
| CTC-228N24.2 | 4.17E-06 | 1.79E-05 | 0.401112 | 0.038226 | 3.625304 |
| CTC-260E6.6 | 8.26E-17 | 2.21E-15 | 0.446284 | 0.143827 | 2.240454 |
| CTC-261N6.1 | 1.35E-15 | 3.03E-14 | 0.335488 | 0.017689 | 3.700318 |
| CTC-268N12.2 | 6.68E-19 | 2.50E-17 | 0.250663 | 0.062818 | 2.153708 |
| CTC-338M12.9 | 5.15E-22 | 3.01E-20 | 0.446265 | 0.029228 | 4.227139 |
| CTC-450M9.1 | 6.86E-13 | 1.00E-11 | 1.226893 | 0.334373 | 3.054733 |
| CTC-453G23.5 | 0.00017 | 0.000513 | 0.24908 | 0.043733 | 2.428667 |
| CTC-455F18.1 | 1.19E-07 | 6.99E-07 | 1.231836 | 0.131814 | 5.745269 |
| CTC-480C2.1 | 7.09E-20 | 3.00E-18 | 0.161915 | 0.000966 | 2.594105 |
| CTC-490G23.2 | 0.005375 | 0.010573 | 9.713121 | 4.201169 | 2.281501 |
| CTC-499J9.1 | 2.48E-13 | 3.89E-12 | 0.215644 | 0.032552 | 2.381286 |
| CTC-524C5.2 | 9.94E-14 | 1.63E-12 | 1.117301 | 0.452534 | 2.203124 |
| CTC-529I10.1 | 2.52E-12 | 3.34E-11 | 1.30981 | 0.555611 | 2.150377 |
| CTC-529I10.2 | 1.45E-14 | 2.71E-13 | 1.690392 | 0.731103 | 2.154237 |
| CTC-542B22.2 | 1.32E-10 | 1.33E-09 | 0.624102 | 0.255567 | 2.036471 |
| CTD-2006H14.2 | 1.45E-10 | 1.44E-09 | 2.057952 | 0.895724 | 2.16722 |
| CTD-2008A1.3 | 1.16E-13 | 1.91E-12 | 0.276917 | 0.046532 | 2.572261 |
| CTD-2008P7.8 | 0.003974 | 0.008131 | 0.115882 | 0.004245 | 2.070908 |
| CTD-2015A6.2 | 8.42E-06 | 3.39E-05 | 0.163077 | 0.014449 | 2.298635 |
| CTD-2015H3.1 | 2.13E-12 | 2.88E-11 | 0.125335 | 0.001221 | 2.22616 |
| CTD-2015H3.2 | 1.77E-13 | 2.82E-12 | 0.109058 | 0.004306 | 2.004273 |
| CTD-2017F17.2 | 1.23E-16 | 3.24E-15 | 3.065305 | 0.770701 | 3.635352 |
| CTD-2021H9.3 | 8.56E-15 | 1.64E-13 | 1.286934 | 0.131742 | 5.98483 |
| CTD-2023M8.1 | 1.14E-10 | 1.15E-09 | 0.734395 | 0.195599 | 2.822731 |
| CTD-2035E11.5 | 1.01E-11 | 1.22E-10 | 0.581486 | 0.211398 | 2.188471 |
| CTD-2066L21.3 | 1.56E-16 | 4.03E-15 | 0.273659 | 0.013486 | 3.292553 |
| CTD-2105E13.15 | 3.06E-13 | 4.71E-12 | 0.40509 | 0.115431 | 2.344557 |
| CTD-2116N20.1 | 5.03E-16 | 1.21E-14 | 0.349239 | 0.066346 | 2.700629 |
| CTD-2147F2.1 | 1.90E-10 | 1.84E-09 | 1.642996 | 0.030011 | 13.40655 |
| CTD-2147F2.2 | 4.63E-09 | 3.57E-08 | 0.261985 | 0.020934 | 2.993242 |
| CTD-2196E14.5 | 2.24E-10 | 2.14E-09 | 0.399113 | 0.125956 | 2.208898 |
| CTD-2199O4.6 | 1.21E-14 | 2.28E-13 | 0.413564 | 0.133582 | 2.198639 |
| CTD-2201I18.1 | 2.50E-10 | 2.38E-09 | 0.323526 | 0.101615 | 2.10067 |
| CTD-2207A17.1 | 0.000385 | 0.00105 | 0.159347 | 0.003318 | 2.510175 |
| CTD-2210P24.3 | 0.007995 | 0.014977 | 0.332696 | 0.015167 | 3.757121 |
| CTD-2227E11.1 | 2.46E-08 | 1.65E-07 | 0.999697 | 0.383464 | 2.274623 |
| CTD-2280E9.1 | 3.29E-16 | 8.14E-15 | 0.172031 | 0.012988 | 2.407604 |
| CTD-2284J15.1 | 2.03E-21 | 1.08E-19 | 0.819061 | 0.24845 | 2.637568 |
| CTD-2288O8.1 | 2.79E-13 | 4.33E-12 | 0.445701 | 0.074118 | 3.134082 |
| CTD-2291D10.4 | 0.000113 | 0.000353 | 0.363513 | 0.122748 | 2.080888 |
| CTD-2331H12.7 | 4.93E-28 | 6.50E-26 | 0.69615 | 0.166432 | 2.988188 |
| CTD-2349P21.11 | 5.16E-18 | 1.68E-16 | 0.197524 | 0.040411 | 2.118951 |
| CTD-2349P21.5 | 1.28E-09 | 1.08E-08 | 0.572946 | 0.232593 | 2.023335 |
| CTD-2349P21.6 | 3.98E-10 | 3.65E-09 | 0.472209 | 0.186002 | 2.000717 |
| CTD-2354A18.1 | 0.002928 | 0.006229 | 1.248879 | 0.007439 | 12.55488 |
| CTD-2357A8.3 | 2.50E-14 | 4.50E-13 | 0.435002 | 0.070937 | 3.129814 |
| CTD-2371O3.3 | 1.46E-17 | 4.48E-16 | 0.788525 | 0.282068 | 2.325569 |
| CTD-2373N4.5 | 5.61E-12 | 7.00E-11 | 0.309717 | 0.09583 | 2.092213 |
| CTD-2377D24.4 | 2.84E-11 | 3.21E-10 | 1.123856 | 0.089265 | 6.466361 |
| CTD-2377D24.6 | 1.58E-18 | 5.58E-17 | 2.274697 | 0.227145 | 7.258855 |
| CTD-2384A14.1 | 4.32E-09 | 3.36E-08 | 0.121493 | 0.008222 | 2.046659 |
| CTD-2385L22.1 | 5.06E-07 | 2.65E-06 | 1.086119 | 0.442402 | 2.186788 |
| CTD-2510F5.4 | 4.32E-32 | 8.63E-30 | 9.200613 | 2.331929 | 3.824377 |
| CTD-2515C13.2 | 0.000524 | 0.001376 | 0.105937 | 0.001952 | 2.019932 |
| CTD-2517M22.17 | 1.19E-18 | 4.25E-17 | 0.642983 | 0.217463 | 2.340376 |
| CTD-2525I3.5 | 6.67E-13 | 9.81E-12 | 0.852066 | 0.313091 | 2.304737 |
| CTD-2527I21.14 | 5.67E-14 | 9.77E-13 | 0.773464 | 0.311414 | 2.12308 |
| CTD-2527I21.15 | 5.39E-15 | 1.07E-13 | 0.808531 | 0.215202 | 2.882375 |
| CTD-2532K18.2 | 3.03E-14 | 5.38E-13 | 0.44453 | 0.002825 | 5.29568 |
| CTD-2535I10.1 | 1.42E-06 | 6.79E-06 | 0.125471 | 0.002066 | 2.209068 |
| CTD-2537I9.12 | 2.13E-21 | 1.13E-19 | 2.801538 | 1.041079 | 2.542803 |
| CTD-2540F13.2 | 5.19E-30 | 8.73E-28 | 1.014259 | 0.152426 | 4.414198 |
| CTD-2587H19.3 | 1.10E-15 | 2.49E-14 | 0.566559 | 0.207181 | 2.169923 |
| CTD-2589H19.6 | 2.56E-21 | 1.32E-19 | 1.576141 | 0.394259 | 3.391219 |
| CTD-2591A6.2 | 8.68E-17 | 2.32E-15 | 0.335915 | 0.002139 | 4.267877 |
| CTD-2616J11.3 | 2.48E-09 | 2.00E-08 | 0.323493 | 0.084611 | 2.293972 |
| CTD-3032J10.4 | 2.78E-08 | 1.84E-07 | 1.645827 | 0.709613 | 2.156373 |
| CTD-3035D6.2 | 3.43E-21 | 1.74E-19 | 0.604236 | 0.079861 | 3.915453 |
| CTD-3051D23.4 | 4.68E-14 | 8.15E-13 | 0.487209 | 0.134375 | 2.505422 |
| CTD-3080P12.3 | 1.20E-05 | 4.66E-05 | 0.560779 | 0.148237 | 2.66189 |
| CTD-3184A7.4 | 1.09E-15 | 2.47E-14 | 3.720597 | 1.698514 | 2.124308 |
| CTD-3195I5.4 | 2.53E-17 | 7.32E-16 | 0.293691 | 0.066389 | 2.366083 |
| CTD-3214H19.6 | 3.90E-11 | 4.30E-10 | 0.436789 | 0.158023 | 2.080391 |
| CYP4A22-AS1 | 4.10E-32 | 8.31E-30 | 0.416772 | 0.05962 | 3.237516 |
| DBH-AS1 | 4.33E-11 | 4.73E-10 | 0.446902 | 0.165264 | 2.061727 |
| DCUN1D2-AS | 8.41E-12 | 1.03E-10 | 0.506567 | 0.176192 | 2.196179 |
| DDX11-AS1 | 2.82E-33 | 6.67E-31 | 0.321816 | 0.092593 | 2.190191 |
| DLEU1-AS1 | 7.74E-43 | 8.24E-40 | 0.129434 | 0.005482 | 2.175096 |
| DLEU2 | 1.37E-36 | 5.38E-34 | 1.592363 | 0.422942 | 3.236234 |
| DLEU7-AS1 | 8.80E-42 | 7.50E-39 | 0.181021 | 0.022846 | 2.287584 |
| DLGAP1-AS2 | 3.34E-40 | 2.25E-37 | 1.996486 | 0.341149 | 4.752332 |
| DLGAP1-AS5 | 0.000136 | 0.000418 | 1.271016 | 0.250126 | 3.91578 |
| DUXAP8 | 1.56E-42 | 1.53E-39 | 1.105172 | 0.071312 | 7.03494 |
| EIF1AX-AS1 | 2.86E-12 | 3.76E-11 | 0.34075 | 0.111945 | 2.079546 |
| ELFN1-AS1 | 1.60E-25 | 1.53E-23 | 5.085259 | 0.602014 | 7.386266 |
| EVX1-AS | 2.77E-08 | 1.84E-07 | 0.120256 | 0.003505 | 2.12797 |
| FAM201A | 5.02E-12 | 6.35E-11 | 0.777563 | 0.320153 | 2.088677 |
| FAM95C | 2.68E-11 | 3.04E-10 | 0.37816 | 0.089715 | 2.520405 |
| FBXL19-AS1 | 1.03E-34 | 3.14E-32 | 1.037229 | 0.427693 | 2.155095 |
| FEZF1-AS1 | 2.04E-41 | 1.63E-38 | 1.824555 | 0.056522 | 12.29578 |
| FIRRE | 2.44E-36 | 9.16E-34 | 0.393564 | 0.030727 | 3.775531 |
| FLJ22447 | 0.001043 | 0.002524 | 0.352948 | 0.079978 | 2.516692 |
| FOXD2-AS1 | 4.68E-50 | 1.20E-46 | 2.897241 | 0.440504 | 5.545274 |
| FOXP4-AS1 | 4.27E-13 | 6.44E-12 | 3.334947 | 1.042628 | 3.006181 |
| FRGCA | 1.97E-10 | 1.91E-09 | 0.250155 | 0.033018 | 2.63239 |
| FSIP2-AS1 | 1.78E-19 | 7.25E-18 | 0.193334 | 0.038151 | 2.123282 |
| GABPB1-AS1 | 1.71E-14 | 3.15E-13 | 1.012338 | 0.424136 | 2.122233 |
| GAPLINC | 1.29E-23 | 9.35E-22 | 0.653147 | 0.114464 | 3.511757 |
| GAS6-AS1 | 4.05E-10 | 3.71E-09 | 0.941223 | 0.364213 | 2.242984 |
| GAS8-AS1 | 1.81E-13 | 2.87E-12 | 0.393893 | 0.117149 | 2.274444 |
| GATA2-AS1 | 2.04E-20 | 9.43E-19 | 1.191977 | 0.377209 | 2.707361 |
| GK-AS1 | 9.41E-06 | 3.75E-05 | 0.723581 | 0.262204 | 2.273801 |
| GLIS3-AS1 | 5.08E-09 | 3.88E-08 | 0.269869 | 0.064649 | 2.246412 |
| GS1-293C5.1 | 2.12E-22 | 1.32E-20 | 0.349364 | 0.070275 | 2.639056 |
| GS1-600G8.5 | 0.000199 | 0.000588 | 0.257255 | 0.010795 | 3.22448 |
| H19 | 2.66E-07 | 1.46E-06 | 44.04127 | 5.772194 | 7.516998 |
| HAGLR | 1.35E-08 | 9.53E-08 | 1.557367 | 0.673246 | 2.143389 |
| HAGLROS | 3.12E-08 | 2.05E-07 | 0.703729 | 0.235671 | 2.394392 |
| HAR1B | 3.50E-07 | 1.88E-06 | 0.7931 | 0.264803 | 2.448172 |
| HLA-DQB1-AS1 | 1.36E-07 | 7.92E-07 | 2.316474 | 1.073783 | 2.058706 |
| HMGA1P4 | 1.11E-15 | 2.51E-14 | 1.801752 | 0.646925 | 2.546107 |
| HNF1A-AS1 | 3.22E-20 | 1.43E-18 | 7.142016 | 1.77645 | 3.859424 |
| HOTAIR | 4.09E-27 | 4.85E-25 | 1.247321 | 0.020119 | 11.21652 |
| HOTTIP | 5.82E-19 | 2.18E-17 | 1.340907 | 0.126859 | 6.351547 |
| HOXA10-AS | 7.49E-39 | 4.35E-36 | 0.657133 | 0.028131 | 5.909035 |
| HOXA11-AS | 4.67E-25 | 4.17E-23 | 1.232897 | 0.077808 | 7.496256 |
| HOXB-AS4 | 1.19E-16 | 3.15E-15 | 1.744585 | 0.24485 | 5.348948 |
| HOXC13-AS | 3.45E-15 | 7.10E-14 | 0.228211 | 0.009398 | 3.000145 |
| HOXC-AS1 | 3.56E-37 | 1.63E-34 | 0.980821 | 0.058672 | 6.811665 |
| HOXC-AS2 | 2.74E-63 | 3.50E-59 | 0.520114 | 0.014663 | 5.408129 |
| HOXC-AS3 | 1.81E-27 | 2.22E-25 | 0.334155 | 0.002154 | 4.249996 |
| HRAT92 | 4.73E-18 | 1.55E-16 | 0.3582 | 0.105118 | 2.233835 |
| IGBP1-AS1 | 4.04E-13 | 6.12E-12 | 0.328465 | 0.113144 | 2.010213 |
| IGF2BP2-AS1 | 1.06E-23 | 7.80E-22 | 0.242224 | 0.046634 | 2.333861 |
| KB-1205A7.2 | 2.78E-11 | 3.14E-10 | 0.261 | 0.069235 | 2.133122 |
| KB-1615E4.2 | 1.49E-10 | 1.48E-09 | 0.268501 | 0.023637 | 2.980518 |
| KB-1732A1.1 | 7.72E-06 | 3.14E-05 | 0.975194 | 0.434258 | 2.0125 |
| KB-1991G8.1 | 4.76E-13 | 7.15E-12 | 0.261689 | 0.067568 | 2.158458 |
| KB-431C1.5 | 2.40E-16 | 6.05E-15 | 0.702305 | 0.25652 | 2.250378 |
| KB-68A7.2 | 1.89E-12 | 2.57E-11 | 0.228079 | 0.04026 | 2.339076 |
| KCNMB2-AS1 | 2.97E-25 | 2.73E-23 | 0.915207 | 0.021533 | 8.353336 |
| KCNQ1OT1 | 8.80E-18 | 2.77E-16 | 0.526929 | 0.140355 | 2.608347 |
| KDM4A-AS1 | 2.00E-19 | 8.13E-18 | 0.482803 | 0.178008 | 2.096356 |
| KIAA0196-AS1 | 9.23E-30 | 1.49E-27 | 0.367962 | 0.072279 | 2.716308 |
| KIF25-AS1 | 2.48E-07 | 1.36E-06 | 0.194951 | 0.039896 | 2.108355 |
| LA16c-306A4.1 | 2.18E-21 | 1.15E-19 | 0.655516 | 0.225297 | 2.322544 |
| LA16c-321D4.2 | 0.000219 | 0.000638 | 0.63778 | 0.230811 | 2.230217 |
| LA16c-325D7.1 | 1.35E-25 | 1.32E-23 | 2.252481 | 0.34234 | 5.318269 |
| LA16c-358B7.3 | 1.59E-17 | 4.84E-16 | 0.779418 | 0.330183 | 2.04429 |
| LA16c-361A3.3 | 4.79E-13 | 7.18E-12 | 1.118928 | 0.429941 | 2.300119 |
| LA16c-390E6.4 | 3.06E-22 | 1.85E-20 | 1.169452 | 0.356938 | 2.778174 |
| LA16c-444G7.2 | 0.000403 | 0.001093 | 0.154173 | 0.021069 | 2.099403 |
| LACTB2-AS1 | 2.36E-28 | 3.17E-26 | 0.252784 | 0.054043 | 2.29016 |
| LENG8-AS1 | 4.72E-16 | 1.14E-14 | 2.925827 | 1.313685 | 2.140382 |
| LHFPL3-AS2 | 1.51E-06 | 7.20E-06 | 2.631306 | 0.923906 | 2.667535 |
| LINC00106 | 2.77E-19 | 1.10E-17 | 1.831011 | 0.626847 | 2.656694 |
| LINC00114 | 0.000752 | 0.001886 | 0.27734 | 0.040196 | 2.691509 |
| LINC00152 | 1.67E-11 | 1.94E-10 | 3.640962 | 1.468512 | 2.385039 |
| LINC00174 | 2.43E-18 | 8.35E-17 | 2.254123 | 0.90326 | 2.346474 |
| LINC00200 | 0.003935 | 0.008062 | 0.121222 | 0.005694 | 2.09304 |
| LINC00205 | 1.44E-16 | 3.75E-15 | 1.830286 | 0.852036 | 2.027535 |
| LINC00221 | 0.000121 | 0.000376 | 0.174524 | 0.014389 | 2.399904 |
| LINC00346 | 9.24E-26 | 9.45E-24 | 0.792354 | 0.240512 | 2.620626 |
| LINC00355 | 2.44E-17 | 7.10E-16 | 0.410976 | 0.009197 | 4.679405 |
| LINC00392 | 0.00045 | 0.001206 | 4.269793 | 0.034738 | 32.43181 |
| LINC00393 | 1.17E-05 | 4.56E-05 | 0.379502 | 0.032646 | 3.614913 |
| LINC00460 | 5.13E-08 | 3.24E-07 | 0.650005 | 0.055512 | 4.822814 |
| LINC00462 | 0.008973 | 0.016529 | 0.354717 | 0.121475 | 2.053128 |
| LINC00494 | 3.79E-11 | 4.19E-10 | 0.448579 | 0.111589 | 2.592668 |
| LINC00501 | 1.06E-14 | 2.02E-13 | 0.319538 | 0.052476 | 2.751496 |
| LINC00508 | 0.002921 | 0.006217 | 0.108112 | 0.002153 | 2.037265 |
| LINC00524 | 8.78E-12 | 1.07E-10 | 0.382823 | 0.034266 | 3.596004 |
| LINC00540 | 8.05E-08 | 4.89E-07 | 0.509453 | 0.06566 | 3.678933 |
| LINC00605 | 7.74E-15 | 1.50E-13 | 0.46596 | 0.092587 | 2.938719 |
| LINC00624 | 1.36E-20 | 6.45E-19 | 0.228248 | 0.060282 | 2.047939 |
| LINC00648 | 2.02E-08 | 1.37E-07 | 0.376241 | 0.023458 | 3.8575 |
| LINC00659 | 2.00E-08 | 1.37E-07 | 2.47648 | 0.628514 | 3.536625 |
| LINC00665 | 1.05E-15 | 2.39E-14 | 3.489611 | 1.17019 | 2.826042 |
| LINC00668 | 0.018033 | 0.030335 | 1.948545 | 0.911874 | 2.024506 |
| LINC00685 | 2.34E-08 | 1.58E-07 | 1.064439 | 0.476924 | 2.018358 |
| LINC00853 | 4.67E-19 | 1.78E-17 | 0.998575 | 0.367589 | 2.349447 |
| LINC00857 | 1.49E-11 | 1.75E-10 | 3.673199 | 1.63448 | 2.175406 |
| LINC00858 | 1.37E-36 | 5.38E-34 | 0.408581 | 0.015363 | 4.408509 |
| LINC00884 | 1.13E-24 | 9.36E-23 | 0.838447 | 0.278697 | 2.478097 |
| LINC00920 | 1.67E-20 | 7.83E-19 | 1.101453 | 0.295192 | 3.040174 |
| LINC00941 | 2.18E-17 | 6.43E-16 | 1.407431 | 0.10997 | 7.179275 |
| LINC00954 | 9.22E-13 | 1.32E-11 | 0.360223 | 0.096043 | 2.347561 |
| LINC00973 | 0.008258 | 0.015409 | 0.441191 | 0.005469 | 5.131267 |
| LINC01004 | 2.21E-24 | 1.76E-22 | 1.581054 | 0.589805 | 2.437 |
| LINC01012 | 6.10E-25 | 5.27E-23 | 0.29631 | 0.078268 | 2.223113 |
| LINC01021 | 4.58E-16 | 1.11E-14 | 0.586751 | 0.026175 | 5.442844 |
| LINC01050 | 1.78E-20 | 8.26E-19 | 0.247971 | 0.00121 | 3.438112 |
| LINC01094 | 2.28E-15 | 4.92E-14 | 0.728624 | 0.231084 | 2.502761 |
| LINC01106 | 3.84E-13 | 5.85E-12 | 0.970042 | 0.344081 | 2.409565 |
| LINC01123 | 1.06E-11 | 1.27E-10 | 0.792161 | 0.275963 | 2.373003 |
| LINC01139 | 0.000164 | 0.000495 | 0.509284 | 0.13583 | 2.583575 |
| LINC01146 | 4.74E-16 | 1.14E-14 | 0.335044 | 0.029631 | 3.356022 |
| LINC01169 | 6.58E-07 | 3.35E-06 | 0.598117 | 0.213613 | 2.22605 |
| LINC01176 | 3.04E-14 | 5.40E-13 | 1.158103 | 0.414039 | 2.447485 |
| LINC01194 | 2.93E-12 | 3.83E-11 | 0.235342 | 0.003684 | 3.234265 |
| LINC01210 | 2.14E-14 | 3.89E-13 | 0.114458 | 0.003764 | 2.066788 |
| LINC01224 | 1.64E-08 | 1.14E-07 | 1.467368 | 0.527298 | 2.498601 |
| LINC01232 | 1.08E-16 | 2.87E-15 | 1.568769 | 0.626158 | 2.298082 |
| LINC01234 | 5.92E-21 | 2.90E-19 | 0.998074 | 0.008509 | 10.11964 |
| LINC01235 | 2.30E-34 | 6.11E-32 | 0.855761 | 0.098409 | 4.817135 |
| LINC01257 | 0.001254 | 0.00297 | 0.106144 | 0.002065 | 2.019725 |
| LINC01260 | 2.46E-05 | 8.88E-05 | 0.747262 | 0.263951 | 2.327953 |
| LINC01270 | 2.79E-13 | 4.33E-12 | 0.35462 | 0.088126 | 2.416572 |
| LINC01272 | 2.22E-33 | 5.46E-31 | 2.292798 | 0.515126 | 3.889933 |
| LINC01287 | 0.021052 | 0.034745 | 0.449574 | 0.007626 | 5.10632 |
| LINC01291 | 5.08E-10 | 4.58E-09 | 1.204121 | 0.467845 | 2.296616 |
| LINC01342 | 7.01E-14 | 1.19E-12 | 0.437266 | 0.12043 | 2.437353 |
| LINC01348 | 3.73E-08 | 2.42E-07 | 0.52167 | 0.141825 | 2.570741 |
| LINC01355 | 2.95E-16 | 7.35E-15 | 1.050756 | 0.373998 | 2.427764 |
| LINC01356 | 6.26E-20 | 2.70E-18 | 0.503124 | 0.096806 | 3.064558 |
| LINC01389 | 4.67E-26 | 4.85E-24 | 1.515599 | 0.352114 | 3.573432 |
| LINC01413 | 0.013007 | 0.022782 | 0.211681 | 0.033867 | 2.328297 |
| LINC01419 | 0.013633 | 0.02379 | 0.300641 | 0.003082 | 3.886635 |
| LINC01436 | 0.000482 | 0.001277 | 1.503847 | 0.678222 | 2.060912 |
| LINC01446 | 3.36E-09 | 2.65E-08 | 0.151482 | 0.013891 | 2.208099 |
| LINC01468 | 0.00546 | 0.010715 | 0.341555 | 0.014503 | 3.856284 |
| LINC01474 | 0.003607 | 0.007478 | 0.330131 | 0.10088 | 2.141234 |
| LINC01503 | 0.000122 | 0.000378 | 2.369227 | 1.085485 | 2.082884 |
| LINC01511 | 0.001321 | 0.003109 | 0.189737 | 0.01267 | 2.571546 |
| LINC01518 | 7.07E-10 | 6.21E-09 | 0.424388 | 0.006481 | 4.924691 |
| LINC01522 | 0.001526 | 0.003539 | 0.72462 | 0.04201 | 5.806756 |
| LINC01549 | 0.014304 | 0.024813 | 0.133542 | 0.012575 | 2.074545 |
| LINC01556 | 1.48E-06 | 7.07E-06 | 0.45627 | 0.175106 | 2.022021 |
| LINC01558 | 8.50E-22 | 4.78E-20 | 0.58974 | 0.132502 | 2.966594 |
| LINC01593 | 6.48E-13 | 9.54E-12 | 0.278942 | 0.030048 | 2.913864 |
| LINC01594 | 5.27E-12 | 6.62E-11 | 0.488866 | 0.050753 | 3.906159 |
| LINC01597 | 2.20E-15 | 4.75E-14 | 0.44647 | 0.116609 | 2.522842 |
| LINC01602 | 0.007677 | 0.014443 | 0.191728 | 0.00858 | 2.686768 |
| LINC01608 | 0.002155 | 0.00479 | 0.11461 | 0.006833 | 2.008829 |
| LINC01611 | 2.25E-14 | 4.08E-13 | 0.215629 | 0.007623 | 2.932733 |
| LINC01612 | 1.27E-05 | 4.88E-05 | 1.026418 | 0.418872 | 2.170896 |
| LINC01614 | 1.57E-28 | 2.18E-26 | 1.558293 | 0.045431 | 11.40264 |
| LINC01615 | 4.02E-20 | 1.76E-18 | 0.407424 | 0.058398 | 3.203476 |
| LINCR-0001 | 3.11E-16 | 7.71E-15 | 0.867396 | 0.122629 | 4.345336 |
| LMO7-AS1 | 1.02E-22 | 6.55E-21 | 0.890135 | 0.253418 | 2.801598 |
| LSAMP-AS1 | 9.76E-11 | 1.01E-09 | 0.388802 | 0.087139 | 2.611971 |
| LUCAT1 | 9.77E-14 | 1.61E-12 | 0.368664 | 0.089647 | 2.47124 |
| LURAP1L-AS1 | 1.18E-14 | 2.24E-13 | 0.203076 | 0.0495 | 2.027259 |
| LVCAT1 | 4.36E-06 | 1.86E-05 | 0.212045 | 0.042183 | 2.194669 |
| MAFA-AS1 | 6.28E-05 | 0.000207 | 0.161244 | 0.027188 | 2.053999 |
| MAFG-AS1 | 1.35E-17 | 4.18E-16 | 2.558969 | 1.024926 | 2.363683 |
| MCCC1-AS1 | 6.83E-10 | 6.02E-09 | 0.420635 | 0.159402 | 2.007062 |
| MCF2L-AS1 | 1.35E-20 | 6.42E-19 | 4.607701 | 1.562975 | 2.83089 |
| MFI2-AS1 | 5.70E-25 | 4.99E-23 | 1.876218 | 0.639355 | 2.672893 |
| MGC32805 | 2.66E-06 | 1.19E-05 | 0.56015 | 0.199746 | 2.202368 |
| MGC39584 | 0.020208 | 0.0335 | 0.215187 | 0.013067 | 2.787606 |
| MIR17HG | 9.34E-25 | 7.96E-23 | 0.541997 | 0.121687 | 2.895959 |
| MIR181A2HG | 7.49E-15 | 1.46E-13 | 0.641416 | 0.222788 | 2.296915 |
| MIR222HG | 3.23E-13 | 4.94E-12 | 1.698959 | 0.629303 | 2.466684 |
| MIR3945HG | 5.63E-15 | 1.11E-13 | 0.544107 | 0.132788 | 2.766919 |
| MIR4435-2HG | 4.26E-51 | 1.36E-47 | 2.588471 | 0.523155 | 4.314291 |
| MIR548XHG | 5.66E-06 | 2.36E-05 | 0.28755 | 0.010983 | 3.491972 |
| MLK7-AS1 | 9.25E-12 | 1.12E-10 | 3.133798 | 0.545856 | 5.006992 |
| MMP25-AS1 | 2.54E-31 | 4.64E-29 | 1.414009 | 0.465218 | 2.678628 |
| MNX1-AS1 | 1.35E-16 | 3.55E-15 | 2.169166 | 0.682041 | 2.901594 |
| MNX1-AS2 | 1.15E-13 | 1.89E-12 | 0.8917 | 0.262309 | 2.73717 |
| MYB-AS1 | 1.39E-23 | 9.97E-22 | 0.153838 | 0.009427 | 2.319704 |
| MYO16-AS1 | 8.80E-06 | 3.53E-05 | 0.252774 | 0.031956 | 2.673421 |
| NAALADL2-AS2 | 0.024426 | 0.039569 | 0.138698 | 0.018327 | 2.017267 |
| NKILA | 2.87E-17 | 8.20E-16 | 1.401804 | 0.303617 | 3.720865 |
| NOVA1-AS1 | 2.45E-11 | 2.79E-10 | 0.225732 | 0.012663 | 2.891204 |
| NPSR1-AS1 | 5.93E-25 | 5.16E-23 | 0.130652 | 0.002737 | 2.245077 |
| NRIR | 5.28E-16 | 1.26E-14 | 0.430802 | 0.099557 | 2.659906 |
| ODF2-AS1 | 7.07E-10 | 6.21E-09 | 0.503167 | 0.199238 | 2.015675 |
| PAX8-AS1 | 7.01E-12 | 8.64E-11 | 1.410362 | 0.546144 | 2.337501 |
| PCAT1 | 1.02E-23 | 7.59E-22 | 0.272209 | 0.054209 | 2.413666 |
| PCAT14 | 0.000459 | 0.001226 | 0.213521 | 0.007236 | 2.923644 |
| PICSAR | 8.75E-05 | 0.00028 | 0.433569 | 0.085594 | 2.874921 |
| POU6F2-AS1 | 0.016474 | 0.028075 | 0.292862 | 0.095849 | 2.005945 |
| POU6F2-AS2 | 2.50E-18 | 8.56E-17 | 0.434482 | 0.011274 | 4.803309 |
| PPP1R26-AS1 | 1.39E-36 | 5.38E-34 | 0.590491 | 0.190242 | 2.379021 |
| PROSER2-AS1 | 6.78E-05 | 0.000222 | 0.276327 | 0.087652 | 2.005451 |
| PRR7-AS1 | 1.51E-32 | 3.28E-30 | 0.662178 | 0.156991 | 2.965773 |
| PTGES2-AS1 | 4.80E-21 | 2.39E-19 | 0.325652 | 0.033711 | 3.183365 |
| PTOV1-AS2 | 9.02E-14 | 1.50E-12 | 2.549562 | 1.143572 | 2.130605 |
| PTPRJ-AS1 | 1.02E-07 | 6.08E-07 | 0.965146 | 0.357951 | 2.325894 |
| PVT1 | 3.45E-11 | 3.84E-10 | 3.439536 | 0.776496 | 4.038278 |
| RHPN1-AS1 | 4.76E-31 | 8.58E-29 | 0.806383 | 0.206746 | 2.954836 |
| RNF144A-AS1 | 8.77E-35 | 2.73E-32 | 0.224362 | 0.037069 | 2.366418 |
| RP1-102E24.8 | 1.10E-23 | 8.05E-22 | 0.713568 | 0.230383 | 2.462498 |
| RP1-102G20.5 | 3.40E-17 | 9.60E-16 | 0.391831 | 0.107741 | 2.367525 |
| RP1-102K2.8 | 2.42E-33 | 5.85E-31 | 0.91999 | 0.149398 | 4.089808 |
| RP11-1008C21.1 | 2.99E-25 | 2.73E-23 | 0.179176 | 0.030133 | 2.145312 |
| RP11-1029J19.4 | 1.72E-20 | 8.01E-19 | 0.418752 | 0.041576 | 3.664118 |
| RP11-1029J19.5 | 4.76E-14 | 8.28E-13 | 0.34022 | 0.069652 | 2.594839 |
| RP11-1038A11.3 | 2.53E-08 | 1.69E-07 | 0.38379 | 0.088965 | 2.560216 |
| RP11-103C16.2 | 8.99E-14 | 1.50E-12 | 0.532778 | 0.170637 | 2.338105 |
| RP11-1041F24.1 | 9.97E-05 | 0.000315 | 0.191482 | 0.00711 | 2.721341 |
| RP11-1055B8.9 | 4.40E-15 | 8.86E-14 | 0.868208 | 0.311207 | 2.354551 |
| RP11-1070N10.5 | 3.45E-06 | 1.51E-05 | 0.171636 | 0.012809 | 2.40792 |
| RP11-1070N10.7 | 7.63E-07 | 3.84E-06 | 0.256599 | 0.021182 | 2.942678 |
| RP11-108K3.1 | 3.77E-17 | 1.06E-15 | 0.588925 | 0.134132 | 2.942467 |
| RP11-108K3.2 | 4.37E-12 | 5.57E-11 | 0.233574 | 0.046074 | 2.283603 |
| RP11-108L7.4 | 5.77E-24 | 4.39E-22 | 0.648795 | 0.171389 | 2.759121 |
| RP11-108M12.3 | 6.06E-14 | 1.04E-12 | 0.137607 | 0.018324 | 2.008107 |
| RP11-109M17.2 | 8.15E-16 | 1.87E-14 | 5.026587 | 0.323313 | 12.11064 |
| RP11-10A14.4 | 1.36E-16 | 3.56E-15 | 0.666222 | 0.140171 | 3.190314 |
| RP11-10A14.5 | 1.18E-08 | 8.47E-08 | 3.497518 | 0.253359 | 10.18093 |
| RP11-10A14.9 | 2.22E-17 | 6.50E-16 | 0.261244 | 0.020433 | 2.999551 |
| RP11-10J21.3 | 1.12E-27 | 1.42E-25 | 0.157637 | 0.017376 | 2.194973 |
| RP11-10J21.4 | 1.72E-34 | 4.79E-32 | 0.401532 | 0.057839 | 3.177496 |
| RP11-10J5.1 | 2.31E-17 | 6.77E-16 | 0.225364 | 0.039334 | 2.335139 |
| RP11-1103G16.1 | 7.93E-23 | 5.17E-21 | 0.229969 | 0.010222 | 2.993679 |
| RP11-111M22.4 | 9.49E-26 | 9.63E-24 | 0.685138 | 0.131939 | 3.385102 |
| RP11-112J3.16 | 2.24E-42 | 2.05E-39 | 0.772392 | 0.17844 | 3.133139 |
| RP11-1143G9.5 | 3.36E-17 | 9.49E-16 | 1.554842 | 0.142248 | 6.831195 |
| RP11-1149O23.2 | 3.56E-20 | 1.57E-18 | 3.342601 | 1.387978 | 2.31361 |
| RP11-1149O23.3 | 2.05E-14 | 3.73E-13 | 1.504777 | 0.626558 | 2.208739 |
| RP11-114G11.5 | 1.27E-06 | 6.13E-06 | 0.228974 | 0.045337 | 2.263527 |
| RP11-115D19.1 | 8.29E-13 | 1.20E-11 | 0.268963 | 0.009091 | 3.382148 |
| RP11-115D19.3 | 2.39E-05 | 8.65E-05 | 0.1514 | 0.022843 | 2.046512 |
| RP11-119B16.2 | 3.17E-15 | 6.62E-14 | 2.136597 | 0.994475 | 2.043534 |
| RP11-11N9.4 | 5.55E-11 | 5.96E-10 | 2.068908 | 0.638879 | 2.935404 |
| RP11-122K13.7 | 3.90E-10 | 3.59E-09 | 0.738094 | 0.246772 | 2.416843 |
| RP11-1260E13.3 | 1.93E-11 | 2.23E-10 | 0.60672 | 0.201024 | 2.347725 |
| RP11-126L15.4 | 3.43E-15 | 7.06E-14 | 1.762345 | 0.569739 | 2.780701 |
| RP11-126O1.4 | 7.47E-10 | 6.52E-09 | 0.2367 | 0.039068 | 2.42112 |
| RP11-1275H24.2 | 9.15E-23 | 5.91E-21 | 0.597039 | 0.15798 | 2.701913 |
| RP11-1275H24.3 | 5.57E-18 | 1.81E-16 | 1.120925 | 0.492835 | 2.059468 |
| RP11-129M16.4 | 1.79E-23 | 1.26E-21 | 1.639863 | 0.511438 | 2.845526 |
| RP11-129M6.1 | 2.97E-09 | 2.37E-08 | 0.451833 | 0.117818 | 2.533458 |
| RP11-131L23.1 | 8.01E-22 | 4.53E-20 | 0.29609 | 0.097705 | 2.00344 |
| RP11-132A1.4 | 2.83E-34 | 7.39E-32 | 3.56614 | 0.768453 | 4.221463 |
| RP11-133N21.10 | 4.57E-11 | 4.99E-10 | 0.31351 | 0.099202 | 2.075834 |
| RP11-134N1.2 | 5.52E-07 | 2.86E-06 | 1.060697 | 0.095625 | 5.93327 |
| RP11-135A1.2 | 3.32E-17 | 9.40E-16 | 0.887543 | 0.229036 | 3.001323 |
| RP11-1376P16.2 | 7.33E-18 | 2.34E-16 | 0.780347 | 0.280033 | 2.316503 |
| RP11-138J23.1 | 5.37E-38 | 2.54E-35 | 0.943622 | 0.009122 | 9.563849 |
| RP11-13J8.1 | 5.50E-14 | 9.50E-13 | 0.375517 | 0.112319 | 2.239631 |
| RP11-13P5.2 | 1.43E-34 | 4.15E-32 | 0.272658 | 0.021953 | 3.055747 |
| RP11-141J13.5 | 0.000266 | 0.000757 | 0.766321 | 0.288696 | 2.22879 |
| RP11-143E21.3 | 0.007507 | 0.014174 | 0.4562 | 0.020587 | 4.612432 |
| RP11-143J12.3 | 5.61E-21 | 2.77E-19 | 0.986409 | 0.314759 | 2.619377 |
| RP11-143K11.7 | 4.38E-12 | 5.59E-11 | 0.677742 | 0.288463 | 2.0021 |
| RP11-145A3.1 | 4.99E-21 | 2.47E-19 | 0.159591 | 0.027281 | 2.039512 |
| RP11-146E13.4 | 1.41E-24 | 1.15E-22 | 0.159767 | 0.012408 | 2.310938 |
| RP11-148K1.12 | 4.51E-26 | 4.73E-24 | 0.733748 | 0.290844 | 2.133198 |
| RP11-150O12.3 | 2.63E-24 | 2.06E-22 | 2.474387 | 0.261346 | 7.124431 |
| RP11-150O12.5 | 5.03E-17 | 1.38E-15 | 0.121438 | 0.01032 | 2.007225 |
| RP11-152H18.4 | 5.76E-17 | 1.57E-15 | 0.252961 | 0.068791 | 2.091117 |
| RP11-152N13.16 | 8.61E-29 | 1.26E-26 | 1.812678 | 0.548173 | 2.950874 |
| RP11-155O18.6 | 3.14E-11 | 3.52E-10 | 0.460676 | 0.179578 | 2.00544 |
| RP11-156K13.3 | 0.000163 | 0.000492 | 0.245614 | 0.019314 | 2.896675 |
| RP11-157P1.4 | 1.97E-08 | 1.35E-07 | 3.097264 | 1.400279 | 2.131112 |
| RP11-159D12.2 | 2.85E-09 | 2.27E-08 | 1.420974 | 0.640989 | 2.052627 |
| RP11-159F24.6 | 5.35E-12 | 6.70E-11 | 0.432024 | 0.153239 | 2.100874 |
| RP11-161I6.2 | 5.75E-23 | 3.81E-21 | 0.184914 | 0.010186 | 2.585745 |
| RP11-161M6.2 | 2.02E-07 | 1.13E-06 | 0.97378 | 0.435002 | 2.007058 |
| RP11-166B2.5 | 2.08E-10 | 2.00E-09 | 0.77502 | 0.326296 | 2.052611 |
| RP11-167H9.4 | 1.13E-18 | 4.04E-17 | 0.294872 | 0.034864 | 2.927919 |
| RP11-167J8.3 | 2.05E-12 | 2.76E-11 | 0.352503 | 0.067059 | 2.708638 |
| RP11-167P22.3 | 4.93E-12 | 6.26E-11 | 0.454521 | 0.125357 | 2.460636 |
| RP11-168F9.2 | 2.34E-31 | 4.34E-29 | 0.979976 | 0.145643 | 4.396532 |
| RP11-168K11.2 | 1.04E-18 | 3.75E-17 | 0.360246 | 0.076781 | 2.603488 |
| RP11-168L7.1 | 0.001531 | 0.003546 | 0.144679 | 0.003292 | 2.368818 |
| RP11-169F17.1 | 8.56E-11 | 8.96E-10 | 0.483659 | 0.026537 | 4.612567 |
| RP11-174G6.1 | 6.27E-06 | 2.59E-05 | 0.606723 | 0.253257 | 2.00059 |
| RP11-175D17.3 | 2.29E-18 | 7.93E-17 | 0.219892 | 0.054594 | 2.069238 |
| RP11-180O5.2 | 0.000599 | 0.001544 | 0.286351 | 0.05994 | 2.415605 |
| RP11-181E10.3 | 5.58E-54 | 3.57E-50 | 0.201458 | 0.019625 | 2.520024 |
| RP11-185E8.1 | 6.16E-08 | 3.83E-07 | 0.299454 | 0.079192 | 2.2292 |
| RP11-186F10.2 | 1.04E-12 | 1.47E-11 | 0.221597 | 0.02088 | 2.660466 |
| RP11-187E13.2 | 6.18E-05 | 0.000204 | 0.453824 | 0.153285 | 2.186561 |
| RP11-18H7.1 | 2.25E-28 | 3.07E-26 | 0.478576 | 0.180689 | 2.061269 |
| RP1-118J21.25 | 9.80E-26 | 9.86E-24 | 0.21301 | 0.042485 | 2.196789 |
| RP11-190J1.3 | 9.06E-39 | 5.03E-36 | 1.443234 | 0.04965 | 10.31227 |
| RP11-191L9.4 | 1.50E-32 | 3.28E-30 | 0.303697 | 0.00413 | 3.876866 |
| RP11-191N8.2 | 2.03E-25 | 1.92E-23 | 0.44801 | 0.050106 | 3.650825 |
| RP11-197K6.1 | 0.006479 | 0.01242 | 0.518715 | 0.002243 | 6.051412 |
| RP11-1C8.7 | 0.012289 | 0.021667 | 0.259956 | 0.069585 | 2.122573 |
| RP11-1M18.1 | 3.30E-16 | 8.17E-15 | 0.203718 | 0.038503 | 2.192863 |
| RP11-206M11.7 | 0.000343 | 0.000951 | 3.871744 | 0.117828 | 18.23341 |
| RP11-20B24.7 | 2.46E-14 | 4.43E-13 | 1.148412 | 0.445847 | 2.287111 |
| RP11-20G13.2 | 3.13E-06 | 1.38E-05 | 0.289945 | 0.06997 | 2.294197 |
| RP11-20G13.3 | 6.46E-08 | 3.99E-07 | 0.406857 | 0.120038 | 2.303494 |
| RP11-20I20.4 | 6.85E-20 | 2.92E-18 | 1.977615 | 0.485253 | 3.549943 |
| RP11-211C9.1 | 2.40E-25 | 2.24E-23 | 0.548883 | 0.049098 | 4.352053 |
| RP11-211G23.2 | 2.49E-21 | 1.29E-19 | 2.329765 | 0.355077 | 5.339237 |
| RP11-212I21.2 | 7.91E-22 | 4.50E-20 | 0.251107 | 0.046016 | 2.40457 |
| RP11-215P8.4 | 3.98E-07 | 2.12E-06 | 0.616219 | 0.054578 | 4.633371 |
| RP11-218F10.3 | 1.08E-22 | 6.93E-21 | 0.679188 | 0.206086 | 2.545649 |
| RP11-219B4.3 | 7.55E-22 | 4.33E-20 | 0.337641 | 0.081863 | 2.40643 |
| RP11-223C24.1 | 1.57E-11 | 1.83E-10 | 0.499006 | 0.097877 | 3.027169 |
| RP11-223C24.2 | 0.001665 | 0.003817 | 0.220787 | 0.040732 | 2.279425 |
| RP11-22C11.2 | 1.23E-21 | 6.82E-20 | 1.185837 | 0.361625 | 2.785457 |
| RP11-22L13.1 | 8.97E-14 | 1.49E-12 | 0.290021 | 0.065946 | 2.350294 |
| RP11-234B24.2 | 0.030418 | 0.047922 | 2.965783 | 1.281204 | 2.219646 |
| RP11-234K24.3 | 1.10E-17 | 3.41E-16 | 0.328919 | 0.100634 | 2.137817 |
| RP11-242D8.1 | 1.04E-24 | 8.71E-23 | 4.694864 | 1.698091 | 2.666641 |
| RP11-242J7.1 | 4.92E-09 | 3.77E-08 | 0.580536 | 0.031938 | 5.158014 |
| RP11-243J16.7 | 1.54E-16 | 3.97E-15 | 0.854864 | 0.255474 | 2.68617 |
| RP11-243J18.2 | 6.25E-09 | 4.70E-08 | 0.423705 | 0.11627 | 2.421536 |
| RP11-244M2.1 | 8.26E-11 | 8.69E-10 | 0.47635 | 0.16192 | 2.200484 |
| RP11-245C17.2 | 5.21E-17 | 1.43E-15 | 0.578433 | 0.166577 | 2.544974 |
| RP11-245P10.4 | 4.48E-14 | 7.85E-13 | 0.917692 | 0.368886 | 2.170445 |
| RP11-253E3.3 | 1.26E-28 | 1.79E-26 | 1.618739 | 0.581834 | 2.52076 |
| RP11-254F7.1 | 3.37E-15 | 6.96E-14 | 0.114029 | 0 | 2.140289 |
| RP11-254F7.4 | 2.58E-17 | 7.43E-16 | 0.449211 | 0.09737 | 2.782644 |
| RP11-256I9.2 | 0.018634 | 0.03121 | 0.16571 | 0.002283 | 2.597787 |
| RP11-259N19.1 | 2.52E-26 | 2.75E-24 | 0.886508 | 0.232812 | 2.964164 |
| RP11-25C19.3 | 7.14E-10 | 6.26E-09 | 0.711204 | 0.241199 | 2.377508 |
| RP11-25H12.1 | 6.05E-16 | 1.43E-14 | 0.173618 | 0.004347 | 2.622192 |
| RP11-260A9.6 | 0.00661 | 0.012645 | 0.164869 | 0.015952 | 2.284294 |
| RP11-260M2.1 | 2.31E-25 | 2.17E-23 | 1.178285 | 0.386067 | 2.629852 |
| RP11-264B17.2 | 1.57E-21 | 8.48E-20 | 0.87368 | 0.322751 | 2.303198 |
| RP11-267A15.3 | 2.15E-20 | 9.85E-19 | 0.128086 | 0.00956 | 2.081835 |
| RP11-267M23.3 | 2.64E-07 | 1.45E-06 | 0.679825 | 0.287624 | 2.011809 |
| RP11-268J15.5 | 3.70E-11 | 4.10E-10 | 1.17003 | 0.457014 | 2.280067 |
| RP11-273B20.1 | 3.63E-23 | 2.46E-21 | 1.326555 | 0.459841 | 2.548144 |
| RP11-276H19.2 | 3.68E-08 | 2.39E-07 | 0.552809 | 0.203895 | 2.148141 |
| RP11-277B15.3 | 5.01E-34 | 1.26E-31 | 0.666327 | 0.164145 | 2.901165 |
| RP11-278H7.4 | 1.42E-08 | 1.00E-07 | 0.152667 | 0.020599 | 2.095104 |
| RP11-279N8.1 | 3.84E-17 | 1.07E-15 | 0.160723 | 0.010807 | 2.352951 |
| RP11-282A11.3 | 1.46E-05 | 5.59E-05 | 0.154837 | 0.016364 | 2.189993 |
| RP11-283G6.3 | 5.41E-07 | 2.81E-06 | 2.114486 | 0.954048 | 2.100935 |
| RP11-283G6.4 | 1.40E-08 | 9.91E-08 | 0.437467 | 0.158594 | 2.078423 |
| RP11-284F21.10 | 1.88E-16 | 4.80E-15 | 5.823992 | 1.631136 | 3.422025 |
| RP11-284F21.7 | 5.60E-07 | 2.89E-06 | 9.000436 | 4.102228 | 2.165621 |
| RP11-284F21.9 | 8.10E-21 | 3.94E-19 | 1.628461 | 0.326057 | 4.056874 |
| RP11-284G10.1 | 3.52E-05 | 0.000123 | 0.233632 | 0.001372 | 3.291178 |
| RP11-285F7.2 | 3.13E-27 | 3.77E-25 | 13.45796 | 4.541855 | 2.920807 |
| RP11-28H5.2 | 2.51E-16 | 6.30E-15 | 0.249021 | 0.067555 | 2.083022 |
| RP11-290D2.3 | 5.54E-11 | 5.96E-10 | 1.234349 | 0.504086 | 2.208873 |
| RP11-290F24.6 | 9.54E-19 | 3.47E-17 | 0.571206 | 0.1762 | 2.430144 |
| RP11-293P20.2 | 1.13E-15 | 2.54E-14 | 0.465204 | 0.089557 | 2.981703 |
| RP11-297C4.1 | 6.12E-08 | 3.81E-07 | 0.293193 | 0.07993 | 2.185261 |
| RP11-297C4.3 | 7.13E-16 | 1.65E-14 | 0.295164 | 0.064861 | 2.396952 |
| RP11-297P16.3 | 2.01E-05 | 7.42E-05 | 0.257246 | 0.002058 | 3.500437 |
| RP11-297P16.4 | 0.000169 | 0.000509 | 11.83829 | 0.047867 | 80.73643 |
| RP11-29H23.4 | 1.85E-12 | 2.52E-11 | 0.299928 | 0.073981 | 2.298695 |
| RP11-302F12.10 | 2.29E-11 | 2.62E-10 | 0.587424 | 0.208389 | 2.229078 |
| RP11-302L19.1 | 1.64E-07 | 9.42E-07 | 0.133642 | 0.003431 | 2.258921 |
| RP11-304F15.3 | 3.39E-21 | 1.72E-19 | 0.407204 | 0.142958 | 2.087617 |
| RP11-304F15.7 | 1.61E-18 | 5.67E-17 | 0.190706 | 0.044442 | 2.012619 |
| RP11-308B16.2 | 1.27E-10 | 1.28E-09 | 0.321641 | 0.011029 | 3.797583 |
| RP11-30L3.2 | 8.92E-09 | 6.55E-08 | 0.289107 | 0.094356 | 2.002033 |
| RP11-310P5.1 | 2.96E-11 | 3.33E-10 | 0.331837 | 0.050971 | 2.860386 |
| RP11-310P5.2 | 0.001626 | 0.003741 | 0.41007 | 0.149444 | 2.044829 |
| RP11-313P13.5 | 1.98E-13 | 3.14E-12 | 0.382017 | 0.138166 | 2.023869 |
| RP11-314A20.2 | 1.23E-14 | 2.31E-13 | 0.575353 | 0.175196 | 2.454077 |
| RP11-316M1.3 | 3.45E-10 | 3.21E-09 | 0.328095 | 0.097414 | 2.168514 |
| RP11-317N12.1 | 0.000366 | 0.001004 | 0.207913 | 0.028942 | 2.387991 |
| RP11-318G21.4 | 0.000437 | 0.001177 | 0.381423 | 0.002942 | 4.676649 |
| RP11-320G10.1 | 2.40E-19 | 9.62E-18 | 0.483235 | 0.021513 | 4.799792 |
| RP11-320G24.1 | 2.11E-05 | 7.76E-05 | 0.190404 | 0.024883 | 2.325419 |
| RP11-322D14.2 | 2.14E-12 | 2.88E-11 | 0.327313 | 0.094304 | 2.199201 |
| RP11-324E6.10 | 4.60E-09 | 3.55E-08 | 0.338352 | 0.107678 | 2.110726 |
| RP11-326C3.2 | 1.89E-21 | 1.01E-19 | 7.376008 | 1.324156 | 5.249431 |
| RP11-328J2.1 | 1.95E-06 | 9.00E-06 | 0.117834 | 0.005024 | 2.074133 |
| RP11-328K4.1 | 8.07E-16 | 1.86E-14 | 0.36296 | 0.025968 | 3.675221 |
| RP11-332K15.1 | 2.02E-13 | 3.20E-12 | 0.211985 | 0.003389 | 3.017589 |
| RP11-334C17.5 | 4.77E-19 | 1.81E-17 | 0.767237 | 0.264419 | 2.379783 |
| RP11-336K24.5 | 2.39E-05 | 8.65E-05 | 0.565141 | 0.185865 | 2.32677 |
| RP11-338N10.3 | 4.34E-18 | 1.43E-16 | 0.518428 | 0.090216 | 3.251194 |
| RP11-33I11.2 | 3.49E-15 | 7.14E-14 | 1.104954 | 0.301773 | 2.999093 |
| RP11-344E13.4 | 5.65E-05 | 0.000188 | 0.13527 | 0.015993 | 2.028305 |
| RP11-344P13.1 | 3.87E-16 | 9.44E-15 | 0.122429 | 0.002694 | 2.165946 |
| RP11-346D19.1 | 1.23E-22 | 7.80E-21 | 0.443485 | 0.009931 | 4.943852 |
| RP11-347C12.11 | 3.31E-20 | 1.47E-18 | 0.973646 | 0.364509 | 2.311355 |
| RP11-350J20.12 | 0.000217 | 0.000633 | 7.082456 | 3.409065 | 2.046829 |
| RP11-351C8.1 | 3.41E-11 | 3.80E-10 | 0.16335 | 0.031167 | 2.007742 |
| RP11-352G18.2 | 1.64E-13 | 2.64E-12 | 0.437338 | 0.159697 | 2.069093 |
| RP11-353N14.1 | 1.26E-21 | 6.95E-20 | 0.165307 | 0.009254 | 2.428347 |
| RP11-353N14.2 | 4.40E-20 | 1.92E-18 | 0.346522 | 0.064658 | 2.711813 |
| RP11-353N14.4 | 7.40E-15 | 1.45E-13 | 1.360067 | 0.450301 | 2.653218 |
| RP11-353N14.5 | 1.80E-39 | 1.15E-36 | 0.459445 | 0.081893 | 3.075688 |
| RP11-357H14.17 | 6.08E-09 | 4.59E-08 | 8.680335 | 2.290765 | 3.672605 |
| RP11-357P18.2 | 5.94E-10 | 5.29E-09 | 0.640817 | 0.254662 | 2.088796 |
| RP11-35G22.1 | 1.48E-11 | 1.74E-10 | 0.622707 | 0.23024 | 2.188431 |
| RP11-362F19.1 | 2.04E-09 | 1.67E-08 | 0.365367 | 0.101801 | 2.306072 |
| RP11-366F6.2 | 5.68E-06 | 2.37E-05 | 0.474265 | 0.000289 | 5.726085 |
| RP11-366H4.1 | 0.002773 | 0.005947 | 0.16786 | 0.002484 | 2.613672 |
| RP11-366L20.2 | 5.23E-28 | 6.75E-26 | 0.321396 | 0.031234 | 3.211019 |
| RP11-370A5.1 | 1.19E-12 | 1.67E-11 | 0.278133 | 0.062344 | 2.329207 |
| RP11-379K22.3 | 0.000626 | 0.001603 | 1.568456 | 0.280436 | 4.385641 |
| RP11-383J24.1 | 1.22E-07 | 7.17E-07 | 0.595563 | 0.083943 | 3.781417 |
| RP11-385F7.1 | 6.49E-15 | 1.28E-13 | 2.428373 | 1.061759 | 2.176332 |
| RP11-388C12.5 | 7.67E-18 | 2.43E-16 | 0.38859 | 0.095158 | 2.503567 |
| RP11-390N6.1 | 1.14E-08 | 8.20E-08 | 0.319867 | 0.029321 | 3.246707 |
| RP11-390P24.1 | 0.00013 | 0.000402 | 4.108514 | 1.559745 | 2.535639 |
| RP11-392O1.4 | 3.28E-10 | 3.06E-09 | 0.24624 | 0.069649 | 2.040914 |
| RP11-394I13.2 | 4.72E-20 | 2.04E-18 | 0.544456 | 0.097036 | 3.270761 |
| RP11-395B7.2 | 0.000494 | 0.001307 | 1.162935 | 0.425585 | 2.402912 |
| RP11-397A16.1 | 8.77E-08 | 5.28E-07 | 0.168285 | 0.003597 | 2.58971 |
| RP11-3B12.5 | 7.22E-10 | 6.33E-09 | 0.298151 | 0.008193 | 3.680006 |
| RP11-400N13.2 | 2.47E-09 | 1.99E-08 | 1.469328 | 0.009794 | 14.29339 |
| RP11-400N13.3 | 2.74E-13 | 4.27E-12 | 1.024445 | 0.152862 | 4.446877 |
| RP11-403P17.6 | 3.80E-16 | 9.28E-15 | 2.019962 | 0.733747 | 2.542692 |
| RP11-408B11.2 | 2.86E-21 | 1.47E-19 | 0.507984 | 0.040671 | 4.322039 |
| RP11-408H1.3 | 5.44E-10 | 4.87E-09 | 0.260684 | 0.079055 | 2.01437 |
| RP1-140K8.5 | 3.65E-05 | 0.000127 | 0.563221 | 0.199455 | 2.214759 |
| RP11-413M3.4 | 3.18E-12 | 4.14E-11 | 0.333529 | 0.106918 | 2.095171 |
| RP11-415F23.4 | 8.50E-13 | 1.22E-11 | 0.747819 | 0.312121 | 2.057208 |
| RP11-415I12.3 | 8.27E-11 | 8.70E-10 | 0.560136 | 0.212987 | 2.109145 |
| RP11-415J8.7 | 1.70E-13 | 2.71E-12 | 0.242841 | 0.066631 | 2.057494 |
| RP11-417E7.2 | 7.45E-13 | 1.09E-11 | 0.552877 | 0.02592 | 5.184841 |
| RP11-419J16.1 | 2.38E-05 | 8.63E-05 | 0.250047 | 0.043984 | 2.431145 |
| RP11-41O4.1 | 1.24E-15 | 2.80E-14 | 0.439045 | 0.162818 | 2.051021 |
| RP11-421F16.3 | 6.79E-16 | 1.59E-14 | 4.47943 | 2.146372 | 2.03859 |
| RP11-426C22.4 | 1.65E-34 | 4.69E-32 | 0.275199 | 0.057604 | 2.38065 |
| RP11-428O18.6 | 4.54E-09 | 3.51E-08 | 0.397112 | 0.070917 | 2.908507 |
| RP11-429J17.5 | 1.71E-24 | 1.37E-22 | 0.609372 | 0.062226 | 4.372731 |
| RP11-429J17.7 | 4.94E-13 | 7.40E-12 | 0.506248 | 0.148764 | 2.437047 |
| RP11-42O4.2 | 3.99E-12 | 5.12E-11 | 0.437835 | 0.126211 | 2.377584 |
| RP11-432I5.2 | 6.28E-09 | 4.72E-08 | 0.375594 | 0.107117 | 2.296255 |
| RP11-432J22.2 | 3.86E-19 | 1.49E-17 | 2.041486 | 0.871196 | 2.204999 |
| RP11-434H6.6 | 1.16E-13 | 1.90E-12 | 0.507924 | 0.18828 | 2.108795 |
| RP11-435O5.4 | 6.36E-13 | 9.39E-12 | 0.407111 | 0.099552 | 2.541242 |
| RP11-435O5.7 | 4.75E-15 | 9.51E-14 | 0.48629 | 0.144822 | 2.394761 |
| RP11-438L19.1 | 3.46E-25 | 3.12E-23 | 0.508801 | 0.161127 | 2.331434 |
| RP11-440D17.3 | 3.84E-17 | 1.07E-15 | 4.025868 | 1.868128 | 2.096341 |
| RP11-440D17.4 | 4.97E-21 | 2.47E-19 | 1.19338 | 0.422981 | 2.473091 |
| RP11-443B20.1 | 7.87E-36 | 2.80E-33 | 1.414589 | 0.326562 | 3.550692 |
| RP11-443B7.1 | 2.32E-06 | 1.05E-05 | 0.892417 | 0.243611 | 2.888203 |
| RP11-443B7.2 | 1.53E-11 | 1.79E-10 | 0.210752 | 0.032308 | 2.348698 |
| RP11-443C10.1 | 2.27E-24 | 1.79E-22 | 0.171464 | 0.021393 | 2.23623 |
| RP11-445O3.2 | 1.68E-08 | 1.17E-07 | 0.139929 | 0 | 2.399287 |
| RP11-449P15.2 | 1.40E-32 | 3.14E-30 | 1.183181 | 0.297813 | 3.225588 |
| RP11-44F14.2 | 1.19E-11 | 1.43E-10 | 1.907851 | 0.648727 | 2.681688 |
| RP11-44F14.8 | 1.61E-13 | 2.60E-12 | 1.188873 | 0.399749 | 2.579042 |
| RP11-44K6.4 | 1.71E-08 | 1.19E-07 | 0.728651 | 0.033912 | 6.188043 |
| RP11-44N12.5 | 1.38E-10 | 1.38E-09 | 0.294104 | 0.088027 | 2.096004 |
| RP11-452H21.1 | 2.32E-07 | 1.29E-06 | 0.289692 | 0.08614 | 2.093536 |
| RP11-456D7.1 | 4.20E-13 | 6.34E-12 | 0.406317 | 0.120884 | 2.292233 |
| RP11-458F8.4 | 6.75E-24 | 5.11E-22 | 4.01257 | 1.291228 | 2.956071 |
| RP11-459O1.2 | 1.05E-05 | 4.14E-05 | 0.322934 | 0.002298 | 4.134324 |
| RP11-462G2.1 | 0.007866 | 0.014763 | 13.18673 | 5.6253 | 2.320704 |
| RP11-463D19.1 | 1.85E-09 | 1.53E-08 | 0.29989 | 0.099069 | 2.008801 |
| RP11-465B22.8 | 2.43E-15 | 5.19E-14 | 6.646634 | 2.20745 | 2.923849 |
| RP11-465N4.5 | 3.80E-22 | 2.26E-20 | 2.889787 | 1.013318 | 2.685475 |
| RP11-467D18.2 | 2.46E-21 | 1.28E-19 | 0.286771 | 0.076828 | 2.187274 |
| RP11-469J4.3 | 2.75E-13 | 4.27E-12 | 0.186085 | 0.04212 | 2.012981 |
| RP11-470P21.2 | 8.34E-13 | 1.20E-11 | 0.354739 | 0.031681 | 3.453352 |
| RP11-473M20.9 | 1.78E-15 | 3.91E-14 | 1.175284 | 0.34508 | 2.865295 |
| RP11-473O4.3 | 8.00E-25 | 6.87E-23 | 0.715947 | 0.188647 | 2.826801 |
| RP11-473O4.4 | 1.97E-14 | 3.60E-13 | 0.470229 | 0.126214 | 2.520754 |
| RP11-473O4.5 | 4.36E-19 | 1.67E-17 | 0.29995 | 0.074621 | 2.290386 |
| RP11-474B12.1 | 1.47E-12 | 2.04E-11 | 0.44623 | 0.154661 | 2.144932 |
| RP11-474D1.3 | 1.21E-08 | 8.66E-08 | 0.46756 | 0.01921 | 4.761017 |
| RP11-474D1.4 | 3.01E-06 | 1.33E-05 | 0.143722 | 0.004549 | 2.331187 |
| RP11-476K15.1 | 2.42E-11 | 2.75E-10 | 0.451295 | 0.003145 | 5.344835 |
| RP11-480A16.1 | 8.19E-27 | 9.61E-25 | 0.571258 | 0.150296 | 2.681861 |
| RP11-481C4.1 | 1.17E-10 | 1.18E-09 | 0.376596 | 0.134349 | 2.033705 |
| RP11-485G7.6 | 1.94E-12 | 2.63E-11 | 0.197346 | 0.027417 | 2.333643 |
| RP11-488L18.10 | 1.14E-24 | 9.44E-23 | 6.091629 | 2.132742 | 2.773105 |
| RP11-492E3.2 | 4.61E-16 | 1.11E-14 | 1.281705 | 0.175442 | 5.016317 |
| RP11-493L12.3 | 2.23E-09 | 1.80E-08 | 0.249001 | 0.04543 | 2.399781 |
| RP11-493L12.5 | 2.45E-07 | 1.35E-06 | 0.434802 | 0.144407 | 2.188165 |
| RP11-495P10.5 | 8.38E-08 | 5.07E-07 | 0.39946 | 0.023736 | 4.036503 |
| RP11-495P10.7 | 6.91E-07 | 3.50E-06 | 1.973449 | 0.127479 | 9.114901 |
| RP11-496H1.2 | 7.42E-18 | 2.35E-16 | 0.879063 | 0.258726 | 2.729277 |
| RP11-496I9.1 | 1.35E-18 | 4.79E-17 | 0.966369 | 0.212455 | 3.412874 |
| RP11-497G19.1 | 8.85E-07 | 4.39E-06 | 0.603475 | 0.039832 | 5.030868 |
| RP11-497G19.2 | 1.94E-05 | 7.18E-05 | 0.469459 | 0.048684 | 3.829996 |
| RP11-497H17.1 | 8.02E-13 | 1.16E-11 | 0.59673 | 0.215834 | 2.206004 |
| RP11-499F3.2 | 1.55E-08 | 1.09E-07 | 0.59409 | 0.100947 | 3.454105 |
| RP11-49K24.4 | 6.65E-11 | 7.09E-10 | 0.259933 | 0.057884 | 2.279735 |
| RP11-4C20.3 | 2.43E-14 | 4.37E-13 | 0.546761 | 0.187143 | 2.252403 |
| RP11-4K16.2 | 5.67E-13 | 8.44E-12 | 0.415284 | 0.108021 | 2.477076 |
| RP11-500G22.5 | 1.50E-11 | 1.76E-10 | 0.914856 | 0.333412 | 2.341552 |
| RP11-501C14.5 | 1.05E-08 | 7.62E-08 | 0.124897 | 0.002875 | 2.186117 |
| RP11-502N13.2 | 7.07E-36 | 2.58E-33 | 0.29327 | 0.036964 | 2.871345 |
| RP11-503E24.2 | 9.83E-13 | 1.39E-11 | 0.989883 | 0.385822 | 2.243378 |
| RP11-504P24.9 | 2.47E-15 | 5.27E-14 | 0.442536 | 0.166689 | 2.034338 |
| RP11-506H20.1 | 2.46E-12 | 3.27E-11 | 0.538517 | 0.205205 | 2.092089 |
| RP11-50C13.1 | 1.23E-27 | 1.54E-25 | 2.232494 | 0.925709 | 2.274032 |
| RP11-519G16.5 | 4.42E-07 | 2.33E-06 | 2.892375 | 1.021919 | 2.667194 |
| RP11-521B24.5 | 1.47E-21 | 8.07E-20 | 2.223178 | 0.715323 | 2.849395 |
| RP11-523H20.3 | 2.03E-19 | 8.19E-18 | 0.928105 | 0.247192 | 2.961198 |
| RP11-523H24.3 | 3.46E-16 | 8.50E-15 | 0.801922 | 0.308637 | 2.207146 |
| RP11-527F13.1 | 1.50E-18 | 5.31E-17 | 0.221741 | 0.053041 | 2.102322 |
| RP11-533O20.2 | 6.33E-07 | 3.23E-06 | 1.617537 | 0.664605 | 2.246307 |
| RP11-534C12.1 | 1.55E-10 | 1.53E-09 | 0.921321 | 0.344849 | 2.295881 |
| RP11-535A5.1 | 0.001317 | 0.003102 | 0.304405 | 0.078925 | 2.260194 |
| RP11-537A6.9 | 4.18E-11 | 4.58E-10 | 0.328466 | 0.113809 | 2.003965 |
| RP11-540O11.6 | 8.17E-11 | 8.61E-10 | 0.424023 | 0.142147 | 2.16407 |
| RP11-540O11.7 | 5.26E-13 | 7.87E-12 | 0.332874 | 0.106448 | 2.09677 |
| RP11-542G1.1 | 2.59E-08 | 1.73E-07 | 0.140414 | 0.012362 | 2.139634 |
| RP11-542K23.9 | 1.93E-06 | 8.93E-06 | 0.271311 | 0.072524 | 2.152227 |
| RP11-542M13.2 | 1.08E-06 | 5.27E-06 | 0.198576 | 0.040983 | 2.117811 |
| RP11-545D19.1 | 0.000637 | 0.001628 | 0.171186 | 0.009574 | 2.474917 |
| RP11-545G3.1 | 0.003011 | 0.006381 | 0.206087 | 0.029418 | 2.365112 |
| RP11-546J1.1 | 2.65E-10 | 2.52E-09 | 0.376998 | 0.105864 | 2.317052 |
| RP11-547D24.1 | 5.16E-29 | 7.94E-27 | 0.168145 | 0.011321 | 2.408753 |
| RP11-548L20.1 | 4.36E-06 | 1.86E-05 | 0.119788 | 0.004812 | 2.096971 |
| RP11-54A9.1 | 1.61E-32 | 3.44E-30 | 0.175217 | 0.017156 | 2.349158 |
| RP11-54H7.4 | 1.53E-07 | 8.84E-07 | 3.423792 | 0.15301 | 13.92749 |
| RP11-54O7.17 | 9.21E-10 | 7.92E-09 | 0.656992 | 0.242874 | 2.207786 |
| RP11-54O7.18 | 2.52E-10 | 2.40E-09 | 0.172057 | 0.030541 | 2.084066 |
| RP11-550A5.2 | 1.28E-15 | 2.87E-14 | 0.436162 | 0.131001 | 2.321038 |
| RP11-553A10.1 | 0.00057 | 0.00148 | 0.29656 | 0.087016 | 2.12046 |
| RP11-554E23.4 | 4.42E-12 | 5.63E-11 | 0.395894 | 0.140383 | 2.062929 |
| RP11-562A8.4 | 3.30E-15 | 6.86E-14 | 0.721609 | 0.259041 | 2.288346 |
| RP11-563K23.1 | 5.35E-07 | 2.78E-06 | 0.191193 | 0.044317 | 2.017726 |
| RP11-567N4.3 | 2.04E-09 | 1.67E-08 | 0.143824 | 0.005888 | 2.302664 |
| RP11-568A7.3 | 3.53E-07 | 1.90E-06 | 0.217555 | 0.053755 | 2.065332 |
| RP11-568J23.5 | 2.66E-16 | 6.67E-15 | 0.511981 | 0.171477 | 2.254268 |
| RP11-575F12.1 | 2.56E-22 | 1.56E-20 | 0.170911 | 0.024555 | 2.17504 |
| RP11-575F12.2 | 1.25E-21 | 6.92E-20 | 1.58888 | 0.358083 | 3.68684 |
| RP11-575F12.3 | 8.45E-28 | 1.08E-25 | 0.914064 | 0.170794 | 3.744776 |
| RP11-57A1.1 | 2.46E-16 | 6.19E-15 | 0.391907 | 0.099743 | 2.462697 |
| RP11-585P4.5 | 2.75E-10 | 2.59E-09 | 1.171887 | 0.482666 | 2.182873 |
| RP11-596D21.1 | 6.61E-17 | 1.79E-15 | 1.060907 | 0.164544 | 4.388336 |
| RP11-598F7.5 | 8.80E-44 | 1.12E-40 | 0.780069 | 0.110644 | 4.177992 |
| RP11-598F7.6 | 1.28E-31 | 2.41E-29 | 0.4948 | 0.0998 | 2.976982 |
| RP11-600F24.7 | 1.98E-25 | 1.89E-23 | 0.513289 | 0.204361 | 2.015006 |
| RP11-611O2.1 | 2.09E-18 | 7.27E-17 | 0.438106 | 0.107343 | 2.595253 |
| RP11-616M22.7 | 5.15E-13 | 7.71E-12 | 0.785797 | 0.096042 | 4.518399 |
| RP11-61O11.1 | 0.028013 | 0.044627 | 0.127273 | 0.012695 | 2.016707 |
| RP11-624L4.1 | 1.44E-17 | 4.40E-16 | 0.326195 | 0.086042 | 2.290859 |
| RP11-626G11.1 | 2.96E-15 | 6.21E-14 | 0.579447 | 0.147311 | 2.747333 |
| RP11-626G11.4 | 4.19E-21 | 2.11E-19 | 0.421386 | 0.096484 | 2.65358 |
| RP11-626G11.5 | 4.90E-12 | 6.22E-11 | 0.328233 | 0.097257 | 2.170938 |
| RP11-626H12.2 | 3.19E-26 | 3.43E-24 | 1.584216 | 0.230665 | 5.093416 |
| RP11-626P14.2 | 1.73E-07 | 9.87E-07 | 0.112746 | 0.003625 | 2.053029 |
| RP11-627G18.2 | 4.08E-17 | 1.13E-15 | 0.648755 | 0.179587 | 2.678072 |
| RP11-62I21.1 | 2.60E-35 | 8.30E-33 | 0.557215 | 0.039148 | 4.72315 |
| RP11-631N16.4 | 2.09E-19 | 8.40E-18 | 1.916537 | 0.666701 | 2.630146 |
| RP11-638I2.9 | 2.77E-07 | 1.52E-06 | 0.946819 | 0.339711 | 2.380699 |
| RP11-640A1.3 | 3.80E-09 | 2.98E-08 | 0.252419 | 0.059469 | 2.209945 |
| RP11-640I15.1 | 3.79E-15 | 7.70E-14 | 0.262791 | 0.075252 | 2.070114 |
| RP11-649A18.12 | 1.21E-24 | 9.89E-23 | 0.62983 | 0.259088 | 2.032452 |
| RP11-655M14.13 | 3.01E-05 | 0.000107 | 1.155062 | 0.266373 | 3.425641 |
| RP11-657O9.1 | 1.31E-14 | 2.46E-13 | 0.200119 | 0.012513 | 2.667405 |
| RP11-65J3.15 | 8.70E-16 | 2.00E-14 | 0.160926 | 0.017637 | 2.218064 |
| RP11-661A12.9 | 5.47E-15 | 1.08E-13 | 0.616344 | 0.127124 | 3.153981 |
| RP11-663N22.1 | 0.000402 | 0.001092 | 0.228298 | 0.017963 | 2.783073 |
| RP11-667M19.10 | 3.01E-09 | 2.39E-08 | 0.597428 | 0.235305 | 2.079979 |
| RP11-669N7.2 | 0.000216 | 0.000632 | 0.939319 | 0.006383 | 9.769634 |
| RP11-672L10.6 | 1.17E-10 | 1.19E-09 | 0.962003 | 0.421393 | 2.036858 |
| RP11-675F6.3 | 8.34E-07 | 4.16E-06 | 0.380225 | 0.065642 | 2.899179 |
| RP11-675F6.4 | 8.25E-07 | 4.12E-06 | 0.292114 | 0.050498 | 2.605449 |
| RP1-167A14.2 | 4.11E-15 | 8.32E-14 | 1.064217 | 0.323298 | 2.750348 |
| RP11-67C2.2 | 1.38E-07 | 8.01E-07 | 0.575206 | 0.235227 | 2.014174 |
| RP11-680A11.5 | 1.39E-22 | 8.77E-21 | 0.799864 | 0.317016 | 2.157863 |
| RP11-680F20.10 | 1.61E-08 | 1.13E-07 | 0.166378 | 0.019307 | 2.232704 |
| RP11-680G24.6 | 1.97E-11 | 2.27E-10 | 1.070285 | 0.440374 | 2.165695 |
| RP11-685N10.1 | 3.79E-19 | 1.47E-17 | 0.808041 | 0.23049 | 2.74756 |
| RP11-69E11.8 | 1.45E-19 | 5.96E-18 | 0.509548 | 0.187905 | 2.117183 |
| RP11-69E9.1 | 4.10E-10 | 3.75E-09 | 0.218409 | 0.043332 | 2.221488 |
| RP11-69G7.1 | 0.000753 | 0.001887 | 0.320494 | 0.011072 | 3.785767 |
| RP11-702H23.4 | 1.80E-15 | 3.95E-14 | 0.245677 | 0.064511 | 2.101243 |
| RP11-703M24.5 | 1.64E-18 | 5.76E-17 | 0.1984 | 0.022118 | 2.443541 |
| RP11-706O15.1 | 1.78E-17 | 5.32E-16 | 2.915805 | 1.293571 | 2.164084 |
| RP11-706O15.3 | 3.38E-08 | 2.20E-07 | 0.489677 | 0.161745 | 2.25287 |
| RP11-707P17.2 | 5.60E-10 | 5.00E-09 | 0.458646 | 0.174884 | 2.032298 |
| RP1-170O19.14 | 3.00E-17 | 8.56E-16 | 1.230109 | 0.147051 | 5.383956 |
| RP1-170O19.17 | 6.62E-08 | 4.08E-07 | 0.13498 | 0.005946 | 2.217928 |
| RP11-713M15.2 | 5.76E-10 | 5.14E-09 | 2.75196 | 0.718433 | 3.484661 |
| RP11-715J22.3 | 1.05E-11 | 1.26E-10 | 0.672284 | 0.236378 | 2.295879 |
| RP11-723O4.9 | 1.28E-06 | 6.16E-06 | 0.993175 | 0.199249 | 3.653066 |
| RP11-727A23.5 | 1.61E-16 | 4.15E-15 | 1.012556 | 0.450117 | 2.022398 |
| RP11-734I18.1 | 0.000457 | 0.001221 | 0.198199 | 0.028445 | 2.321609 |
| RP11-736N17.10 | 4.62E-11 | 5.03E-10 | 0.736088 | 0.189037 | 2.892672 |
| RP11-73M7.1 | 9.13E-30 | 1.49E-27 | 1.901767 | 0.45398 | 3.613426 |
| RP11-73M7.6 | 8.06E-15 | 1.56E-13 | 0.321038 | 0.103557 | 2.068407 |
| RP11-742B18.1 | 8.23E-23 | 5.34E-21 | 0.164284 | 0.002845 | 2.56973 |
| RP11-757F18.5 | 5.73E-19 | 2.16E-17 | 0.939323 | 0.311138 | 2.527919 |
| RP11-758M4.4 | 2.04E-08 | 1.39E-07 | 0.213544 | 0.010863 | 2.828219 |
| RP11-763F8.1 | 4.19E-12 | 5.35E-11 | 0.205658 | 0.013764 | 2.686763 |
| RP11-76C10.5 | 0.003016 | 0.006391 | 0.350637 | 0.079004 | 2.517477 |
| RP11-773H22.4 | 6.05E-18 | 1.94E-16 | 0.613286 | 0.181466 | 2.53418 |
| RP11-774D14.1 | 0.0007 | 0.001769 | 0.210358 | 0.00817 | 2.869171 |
| RP11-776H12.1 | 2.48E-23 | 1.73E-21 | 0.61268 | 0.084692 | 3.858757 |
| RP11-785D18.3 | 0.011696 | 0.020788 | 2.450956 | 0.134259 | 10.88947 |
| RP11-78L16.1 | 1.79E-06 | 8.35E-06 | 0.204529 | 0.004349 | 2.918374 |
| RP11-791G15.2 | 1.71E-16 | 4.40E-15 | 1.139414 | 0.499063 | 2.068921 |
| RP11-794G24.1 | 9.19E-09 | 6.74E-08 | 0.258681 | 0.044251 | 2.486506 |
| RP11-796E10.1 | 8.78E-15 | 1.68E-13 | 1.056452 | 0.215602 | 3.664276 |
| RP11-801I18.1 | 3.28E-06 | 1.44E-05 | 0.109171 | 0 | 2.091712 |
| RP11-802E16.3 | 1.10E-25 | 1.09E-23 | 1.283679 | 0.454925 | 2.493454 |
| RP11-803D5.4 | 1.79E-40 | 1.27E-37 | 0.799368 | 0.087121 | 4.806336 |
| RP11-805L22.3 | 0.002697 | 0.005809 | 0.124832 | 0 | 2.248319 |
| RP11-815M8.1 | 5.20E-22 | 3.02E-20 | 0.25606 | 0.028277 | 2.775712 |
| RP11-81H3.2 | 1.20E-20 | 5.74E-19 | 0.132925 | 0.007953 | 2.15766 |
| RP1-182D15.2 | 1.48E-08 | 1.04E-07 | 0.316213 | 0.099851 | 2.082621 |
| RP11-82O19.1 | 2.39E-09 | 1.93E-08 | 1.137033 | 0.516003 | 2.008162 |
| RP11-83J21.3 | 0.004527 | 0.009124 | 0.149658 | 0.016784 | 2.137776 |
| RP11-84A19.4 | 7.80E-30 | 1.29E-27 | 0.598161 | 0.127777 | 3.065107 |
| RP11-85G21.2 | 9.12E-06 | 3.64E-05 | 0.141686 | 0.002947 | 2.347667 |
| RP11-862P13.1 | 1.70E-09 | 1.41E-08 | 0.664211 | 0.253521 | 2.161712 |
| RP11-863P13.3 | 1.09E-34 | 3.24E-32 | 0.285858 | 0.0253 | 3.079477 |
| RP11-867G2.5 | 4.70E-25 | 4.17E-23 | 0.219697 | 0.007398 | 2.976743 |
| RP11-867G2.6 | 7.67E-19 | 2.83E-17 | 0.314611 | 0.007675 | 3.850583 |
| RP11-874J12.4 | 3.01E-12 | 3.93E-11 | 0.299777 | 0.008022 | 3.700873 |
| RP11-875O11.3 | 3.54E-12 | 4.58E-11 | 0.572913 | 0.18912 | 2.327455 |
| RP11-884K10.6 | 6.41E-29 | 9.76E-27 | 0.611423 | 0.079758 | 3.957669 |
| RP11-88E10.4 | 0.000303 | 0.00085 | 0.310649 | 0.10374 | 2.015552 |
| RP11-88E10.5 | 6.32E-20 | 2.71E-18 | 0.743457 | 0.310304 | 2.055689 |
| RP11-89K21.1 | 8.52E-22 | 4.78E-20 | 1.091072 | 0.031725 | 9.042097 |
| RP11-909N17.2 | 3.05E-09 | 2.42E-08 | 0.159686 | 0.028354 | 2.023207 |
| RP11-91P24.5 | 5.82E-09 | 4.41E-08 | 0.43006 | 0.163488 | 2.011709 |
| RP11-930P14.2 | 3.06E-25 | 2.77E-23 | 0.584581 | 0.228567 | 2.083537 |
| RP11-932O9.10 | 1.37E-23 | 9.90E-22 | 0.599065 | 0.129069 | 3.051761 |
| RP11-93B14.4 | 2.76E-12 | 3.65E-11 | 0.277321 | 0.030058 | 2.901171 |
| RP11-93H24.3 | 1.73E-21 | 9.29E-20 | 1.08382 | 0.364066 | 2.550975 |
| RP11-93K22.13 | 9.36E-14 | 1.55E-12 | 1.06938 | 0.353905 | 2.576265 |
| RP11-93K22.6 | 2.28E-22 | 1.41E-20 | 0.300827 | 0.033514 | 3.00214 |
| RP11-94P11.4 | 1.50E-18 | 5.31E-17 | 0.239196 | 0.01466 | 2.958271 |
| RP11-95I16.6 | 0.008558 | 0.015903 | 2.174426 | 0.903417 | 2.266681 |
| RP11-95M15.1 | 3.57E-15 | 7.27E-14 | 0.400345 | 0.066629 | 3.002743 |
| RP11-962G15.1 | 9.10E-18 | 2.85E-16 | 0.506044 | 0.006892 | 5.669671 |
| RP11-968A15.2 | 2.80E-38 | 1.43E-35 | 0.825367 | 0.170382 | 3.422438 |
| RP11-96D1.5 | 8.23E-10 | 7.15E-09 | 0.522595 | 0.206592 | 2.030693 |
| RP11-973H7.4 | 1.65E-08 | 1.15E-07 | 0.496546 | 0.062813 | 3.664004 |
| RP11-97C16.1 | 2.11E-16 | 5.36E-15 | 3.197929 | 1.385258 | 2.220441 |
| RP11-981G7.6 | 1.11E-17 | 3.45E-16 | 0.455391 | 0.146001 | 2.257675 |
| RP11-98D18.17 | 1.07E-23 | 7.86E-22 | 0.177208 | 0.029615 | 2.138706 |
| RP11-98F14.11 | 5.56E-07 | 2.88E-06 | 0.320786 | 0.091981 | 2.191813 |
| RP1-239B22.5 | 1.93E-23 | 1.36E-21 | 2.392512 | 0.570025 | 3.72003 |
| RP1-267L14.6 | 2.06E-18 | 7.21E-17 | 0.368613 | 0.121987 | 2.110997 |
| RP1-276N6.2 | 0.000416 | 0.001127 | 0.322517 | 0 | 4.225167 |
| RP1-290I10.5 | 0.00151 | 0.003505 | 0.167464 | 0.020855 | 2.213091 |
| RP13-16H11.7 | 3.89E-05 | 0.000134 | 0.173218 | 0.006052 | 2.576269 |
| RP13-455A7.1 | 5.85E-18 | 1.89E-16 | 0.183032 | 0.009746 | 2.578977 |
| RP13-463N16.6 | 0.016672 | 0.028363 | 0.378824 | 0.102309 | 2.366795 |
| RP13-46H24.1 | 3.16E-11 | 3.54E-10 | 0.483683 | 0.105596 | 2.838973 |
| RP13-638C3.3 | 1.79E-15 | 3.92E-14 | 0.49421 | 0.173065 | 2.176077 |
| RP13-714J12.1 | 4.18E-05 | 0.000143 | 0.320543 | 0.10272 | 2.074502 |
| RP13-726E6.1 | 4.54E-08 | 2.90E-07 | 0.345903 | 0.08111 | 2.462052 |
| RP13-890H12.2 | 2.71E-18 | 9.14E-17 | 0.406361 | 0.10922 | 2.42023 |
| RP1-40E16.9 | 0.001874 | 0.004234 | 0.158112 | 0.010518 | 2.335469 |
| RP1-60O19.1 | 0.000542 | 0.001418 | 1.729947 | 0.080521 | 10.13706 |
| RP1-68D18.4 | 4.95E-05 | 0.000167 | 1.311572 | 0.391872 | 2.869792 |
| RP1-78O14.1 | 6.06E-15 | 1.19E-13 | 0.18358 | 0.039731 | 2.029469 |
| RP1-80N2.3 | 3.80E-27 | 4.54E-25 | 0.395566 | 0.119387 | 2.258868 |
| RP1-86C11.7 | 7.93E-16 | 1.83E-14 | 1.154452 | 0.316678 | 3.010606 |
| RP1-86D1.5 | 1.00E-17 | 3.13E-16 | 0.175144 | 0.034705 | 2.042569 |
| RP1-90G24.10 | 1.14E-08 | 8.19E-08 | 0.499247 | 0.07104 | 3.50354 |
| RP1-90G24.11 | 2.04E-10 | 1.97E-09 | 0.305821 | 0.062374 | 2.499297 |
| RP1-90G24.6 | 2.32E-07 | 1.29E-06 | 0.606794 | 0.057776 | 4.479723 |
| RP1-92O14.3 | 9.11E-18 | 2.85E-16 | 1.426417 | 0.637133 | 2.070748 |
| RP3-325F22.5 | 1.76E-08 | 1.22E-07 | 1.983899 | 0.857883 | 2.175526 |
| RP3-337H4.8 | 2.29E-26 | 2.54E-24 | 1.161513 | 0.458488 | 2.258803 |
| RP3-337H4.9 | 4.41E-32 | 8.67E-30 | 1.215732 | 0.310142 | 3.207992 |
| RP3-340N1.2 | 2.26E-10 | 2.16E-09 | 0.507763 | 0.045477 | 4.177733 |
| RP3-355L5.4 | 3.99E-12 | 5.12E-11 | 0.597437 | 0.220224 | 2.177965 |
| RP3-405J10.4 | 8.25E-05 | 0.000266 | 0.921083 | 0.408363 | 2.008572 |
| RP3-406A7.7 | 5.47E-14 | 9.47E-13 | 4.008964 | 1.46925 | 2.618424 |
| RP3-416H24.1 | 6.39E-16 | 1.50E-14 | 1.026778 | 0.134149 | 4.812223 |
| RP3-417L20.4 | 0.001343 | 0.003154 | 0.485277 | 0.084624 | 3.170101 |
| RP3-431P23.5 | 2.62E-18 | 8.89E-17 | 0.368265 | 0.117868 | 2.14931 |
| RP3-449M8.9 | 3.19E-14 | 5.65E-13 | 0.240359 | 0.066367 | 2.045834 |
| RP3-460G2.2 | 3.76E-10 | 3.47E-09 | 0.78261 | 0.212725 | 2.822324 |
| RP3-508I15.21 | 1.42E-22 | 8.93E-21 | 1.969951 | 0.810849 | 2.272551 |
| RP3-522D1.1 | 3.28E-19 | 1.29E-17 | 1.303139 | 0.292043 | 3.579046 |
| RP4-534N18.2 | 3.66E-18 | 1.22E-16 | 0.60376 | 0.190219 | 2.424925 |
| RP4-550H1.7 | 6.90E-12 | 8.51E-11 | 0.362597 | 0.103329 | 2.275119 |
| RP4-563E14.1 | 9.21E-20 | 3.85E-18 | 0.891427 | 0.296276 | 2.50186 |
| RP4-564F22.5 | 2.01E-28 | 2.76E-26 | 0.277855 | 0.055492 | 2.430061 |
| RP4-569M23.5 | 1.45E-24 | 1.17E-22 | 0.347599 | 0.099504 | 2.243561 |
| RP4-583P15.16 | 3.86E-10 | 3.55E-09 | 0.550731 | 0.217281 | 2.050958 |
| RP4-584D14.5 | 3.32E-19 | 1.30E-17 | 1.775325 | 0.760399 | 2.179601 |
| RP4-585I14.3 | 1.07E-24 | 8.97E-23 | 0.231867 | 0.042783 | 2.324273 |
| RP4-594A5.1 | 8.51E-37 | 3.63E-34 | 0.506257 | 0.014281 | 5.30495 |
| RP4-604K5.3 | 2.44E-19 | 9.73E-18 | 0.288409 | 0.069804 | 2.287397 |
| RP4-607I7.1 | 4.14E-05 | 0.000142 | 0.349249 | 0.070066 | 2.641613 |
| RP4-660H19.1 | 1.80E-06 | 8.40E-06 | 0.293924 | 0.064308 | 2.397475 |
| RP4-669H2.1 | 5.27E-10 | 4.73E-09 | 0.26434 | 0.081562 | 2.006698 |
| RP4-680D5.8 | 7.41E-07 | 3.74E-06 | 1.011841 | 0.385624 | 2.289507 |
| RP4-681N20.5 | 5.69E-12 | 7.09E-11 | 0.928884 | 0.406242 | 2.032394 |
| RP4-694A7.2 | 0.001553 | 0.003591 | 0.440141 | 0.034572 | 4.013756 |
| RP4-694B14.8 | 1.75E-13 | 2.79E-12 | 1.48311 | 0.632602 | 2.16094 |
| RP4-724E13.2 | 1.87E-17 | 5.57E-16 | 0.370972 | 0.123757 | 2.104841 |
| RP4-724E16.2 | 1.56E-35 | 5.11E-33 | 0.709279 | 0.172153 | 2.973616 |
| RP4-758J18.13 | 1.80E-26 | 2.03E-24 | 1.319714 | 0.476244 | 2.463739 |
| RP4-760C5.3 | 1.41E-35 | 4.86E-33 | 0.523406 | 0.088352 | 3.309785 |
| RP4-798A10.7 | 5.05E-16 | 1.21E-14 | 0.314754 | 0.069263 | 2.450351 |
| RP5-1009E24.8 | 3.49E-15 | 7.14E-14 | 0.602833 | 0.166053 | 2.641706 |
| RP5-1011O1.2 | 4.40E-21 | 2.20E-19 | 1.436385 | 0.333518 | 3.543992 |
| RP5-1056H1.2 | 2.67E-30 | 4.61E-28 | 1.233562 | 0.330747 | 3.095926 |
| RP5-1061H20.4 | 2.54E-45 | 4.06E-42 | 0.755325 | 0.205078 | 2.803629 |
| RP5-1074L1.4 | 8.07E-29 | 1.20E-26 | 1.422365 | 0.375061 | 3.204564 |
| RP5-1086K13.1 | 9.62E-11 | 9.94E-10 | 0.562536 | 0.196108 | 2.237485 |
| RP5-1092A3.4 | 9.66E-29 | 1.40E-26 | 0.900008 | 0.263973 | 2.747475 |
| RP5-1096J16.1 | 2.25E-05 | 8.19E-05 | 0.289028 | 0.016364 | 3.343196 |
| RP5-1112D6.4 | 4.55E-15 | 9.13E-14 | 2.558527 | 1.061338 | 2.289193 |
| RP5-1120P11.1 | 3.39E-13 | 5.17E-12 | 1.360914 | 0.087688 | 7.783753 |
| RP5-1125A11.4 | 2.92E-20 | 1.32E-18 | 0.283804 | 0.070183 | 2.255249 |
| RP5-1125A11.6 | 1.03E-15 | 2.37E-14 | 1.009189 | 0.370835 | 2.35579 |
| RP5-1125A11.7 | 8.80E-17 | 2.35E-15 | 0.697442 | 0.272291 | 2.141988 |
| RP5-1132H15.1 | 4.82E-15 | 9.62E-14 | 0.730517 | 0.252889 | 2.35348 |
| RP5-1158E12.3 | 9.90E-15 | 1.88E-13 | 1.782405 | 0.467943 | 3.314425 |
| RP5-1184F4.5 | 4.74E-19 | 1.80E-17 | 0.772238 | 0.172978 | 3.195266 |
| RP5-823G15.5 | 1.35E-13 | 2.18E-12 | 0.88902 | 0.098504 | 4.982371 |
| RP5-858B6.1 | 1.28E-09 | 1.08E-08 | 0.188626 | 0.017884 | 2.448379 |
| RP5-881L22.5 | 4.43E-20 | 1.93E-18 | 2.889163 | 0.440169 | 5.533758 |
| RP5-884M6.1 | 1.54E-23 | 1.10E-21 | 0.728149 | 0.036023 | 6.08829 |
| RP5-901A4.1 | 8.99E-24 | 6.76E-22 | 0.7243 | 0.18722 | 2.869923 |
| RP5-907D15.4 | 0.006341 | 0.012189 | 0.308416 | 0.00823 | 3.773593 |
| RP5-908M14.5 | 5.72E-06 | 2.38E-05 | 0.304928 | 0.045539 | 2.782256 |
| RP5-908M14.9 | 1.60E-19 | 6.57E-18 | 2.938442 | 1.385088 | 2.045968 |
| RP5-943J3.2 | 3.12E-20 | 1.40E-18 | 0.616493 | 0.190649 | 2.465147 |
| RP5-963E22.6 | 3.55E-32 | 7.32E-30 | 0.566413 | 0.114883 | 3.101275 |
| RP5-965G21.3 | 3.17E-38 | 1.56E-35 | 0.702974 | 0.197117 | 2.702556 |
| RP5-965G21.4 | 1.94E-20 | 8.99E-19 | 0.655746 | 0.126227 | 3.340656 |
| RP5-965G21.5 | 1.49E-12 | 2.07E-11 | 0.190939 | 0.039701 | 2.082586 |
| RP5-965G21.6 | 1.53E-17 | 4.64E-16 | 2.134604 | 0.752891 | 2.620034 |
| RP5-967N21.11 | 6.39E-32 | 1.22E-29 | 2.133664 | 0.613062 | 3.132497 |
| RP5-991G20.1 | 9.93E-30 | 1.59E-27 | 0.856745 | 0.36327 | 2.065199 |
| RP6-114E22.1 | 1.00E-25 | 9.99E-24 | 0.720929 | 0.017872 | 6.964602 |
| RP6-74O6.6 | 6.85E-07 | 3.47E-06 | 0.739172 | 0.318928 | 2.003143 |
| RUSC1-AS1 | 8.08E-29 | 1.20E-26 | 1.442464 | 0.527685 | 2.457384 |
| SAMD12-AS1 | 4.49E-14 | 7.85E-13 | 0.601445 | 0.208596 | 2.273021 |
| SHANK2-AS2 | 5.88E-13 | 8.73E-12 | 0.254894 | 0.065785 | 2.140694 |
| SLCO4A1-AS1 | 3.20E-09 | 2.53E-08 | 4.561131 | 0.580821 | 6.846335 |
| SMPD5 | 3.33E-18 | 1.11E-16 | 0.381606 | 0.125494 | 2.13578 |
| SNHG1 | 5.49E-18 | 1.79E-16 | 10.9377 | 4.370724 | 2.468885 |
| SNHG12 | 1.31E-15 | 2.92E-14 | 3.488238 | 1.52785 | 2.20428 |
| SNHG15 | 7.73E-19 | 2.84E-17 | 4.521772 | 2.089631 | 2.110754 |
| SNHG17 | 2.98E-13 | 4.60E-12 | 6.896984 | 3.116971 | 2.175022 |
| ST8SIA6-AS1 | 1.76E-08 | 1.22E-07 | 0.88368 | 0.035204 | 7.275533 |
| TFAP2A-AS1 | 2.44E-17 | 7.10E-16 | 0.561622 | 0.183938 | 2.330165 |
| THAP9-AS1 | 3.65E-23 | 2.46E-21 | 5.978314 | 2.620121 | 2.234575 |
| TM4SF1-AS1 | 9.12E-14 | 1.51E-12 | 0.638055 | 0.19837 | 2.473625 |
| TMPO-AS1 | 1.71E-17 | 5.12E-16 | 1.393955 | 0.57348 | 2.21826 |
| TNRC6C-AS1 | 5.11E-12 | 6.45E-11 | 1.289893 | 0.558953 | 2.109243 |
| TRIM31-AS1 | 8.91E-33 | 2.03E-30 | 2.224485 | 0.404928 | 4.603593 |
| TRPM2-AS | 2.85E-43 | 3.31E-40 | 1.926616 | 0.166458 | 7.605764 |
| TSPEAR-AS1 | 1.59E-13 | 2.57E-12 | 0.791311 | 0.169217 | 3.31075 |
| TSPEAR-AS2 | 3.63E-19 | 1.41E-17 | 1.136858 | 0.205663 | 4.046472 |
| TYMSOS | 1.06E-12 | 1.50E-11 | 2.265267 | 1.013903 | 2.123405 |
| UBE2Q1-AS1 | 4.51E-16 | 1.09E-14 | 0.391112 | 0.098041 | 2.479847 |
| UCA1 | 7.27E-08 | 4.44E-07 | 13.09689 | 0.887563 | 13.36308 |
| UTAT33 | 1.40E-14 | 2.62E-13 | 0.331505 | 0.114433 | 2.012307 |
| VPS9D1-AS1 | 2.36E-32 | 4.95E-30 | 6.909482 | 1.516051 | 4.337414 |
| WASIR2 | 1.81E-12 | 2.47E-11 | 0.331392 | 0.107521 | 2.078787 |
| XXbac-B444P24.8 | 1.21E-13 | 1.97E-12 | 0.360285 | 0.063358 | 2.81764 |
| XXbac-BPG252P9.9 | 4.86E-25 | 4.28E-23 | 0.440298 | 0.142437 | 2.228611 |
| XXbac-BPG308K3.5 | 4.77E-12 | 6.07E-11 | 0.184544 | 0.040354 | 2.027334 |
| XXbac-BPG55C20.7 | 6.14E-07 | 3.15E-06 | 0.304692 | 0.073924 | 2.32684 |
| Z69666.2 | 2.08E-09 | 1.69E-08 | 0.536815 | 0.203252 | 2.099949 |
| Z83001.1 | 1.98E-13 | 3.13E-12 | 0.319785 | 0.106103 | 2.036777 |
| ZBTB46-AS1 | 2.75E-06 | 1.23E-05 | 0.616625 | 0.038118 | 5.188481 |
| ZFAS1 | 3.60E-13 | 5.48E-12 | 40.48186 | 19.78309 | 2.041023 |
| ZFPM2-AS1 | 1.30E-12 | 1.82E-11 | 0.689707 | 0.11348 | 3.699213 |
| ZNF252P-AS1 | 1.50E-35 | 5.05E-33 | 0.230047 | 0.062209 | 2.034701 |
| A2M-AS1 | 0.005292 | 0.010433 | 0.492402 | 1.376589 | 0.401197 |
| AC002398.12 | 0.006987 | 0.013278 | 0.382936 | 1.854009 | 0.247152 |
| AC003090.1 | 0.020393 | 0.033759 | 0.343127 | 0.960671 | 0.41778 |
| AC004637.1 | 0.008013 | 0.015003 | 0.234919 | 0.706185 | 0.415437 |
| AC006007.1 | 0.001106 | 0.002663 | 0.061128 | 0.419916 | 0.309911 |
| AC008268.1 | 0.015108 | 0.026051 | 0.077958 | 0.798347 | 0.198095 |
| AC053503.6 | 0.003318 | 0.006942 | 1.1941 | 5.06848 | 0.250383 |
| AC079305.8 | 0.009339 | 0.017143 | 0.169742 | 0.551784 | 0.413852 |
| AC090044.2 | 0.016773 | 0.028508 | 0.199138 | 0.516694 | 0.485068 |
| AC090616.2 | 0.009152 | 0.016828 | 0.328667 | 1.177996 | 0.335421 |
| AC093702.1 | 0.028532 | 0.045324 | 0.22147 | 0.885823 | 0.326093 |
| AC103563.8 | 0.000958 | 0.002339 | 0.178679 | 1.737767 | 0.15164 |
| AC104699.1 | 0.011715 | 0.020813 | 1.074575 | 3.283941 | 0.347103 |
| AC105398.3 | 0.006182 | 0.011932 | 0.031553 | 0.164418 | 0.49752 |
| AC133680.1 | 0.000168 | 0.000506 | 0.134165 | 0.651883 | 0.311438 |
| AC144831.3 | 0.000478 | 0.00127 | 0.545759 | 1.223548 | 0.4879 |
| ADAMTS9-AS1 | 0.001181 | 0.002819 | 0.10605 | 0.604227 | 0.29259 |
| ADAMTS9-AS2 | 0.001608 | 0.003703 | 0.184349 | 0.700383 | 0.355267 |
| AF001548.6 | 0.002661 | 0.00575 | 1.282295 | 5.163658 | 0.262611 |
| AL035610.1 | 0.027567 | 0.044021 | 0.097502 | 0.615992 | 0.275843 |
| AL122127.25 | 0.002708 | 0.005827 | 0.241219 | 0.747415 | 0.402659 |
| AL928768.3 | 0.001864 | 0.004213 | 1.783928 | 20.37301 | 0.09202 |
| AP000345.4 | 0.006698 | 0.012786 | 0.030652 | 0.168995 | 0.485705 |
| AP000692.10 | 0.000498 | 0.001316 | 0.361998 | 0.886016 | 0.46855 |
| AP001347.6 | 0.00567 | 0.011068 | 0.306489 | 0.880581 | 0.414539 |
| ARHGEF26-AS1 | 0.00434 | 0.008794 | 0.069202 | 0.263 | 0.466121 |
| BARX1-AS1 | 0.005209 | 0.010299 | 0.397059 | 1.330054 | 0.347581 |
| BVES-AS1 | 0.000631 | 0.001614 | 0.111913 | 0.404487 | 0.420056 |
| C20orf166-AS1 | 0.001738 | 0.003963 | 0.293825 | 1.566802 | 0.236276 |
| C5orf66-AS1 | 0.003907 | 0.008011 | 1.173604 | 9.142051 | 0.137805 |
| CARMN | 0.017961 | 0.030238 | 1.081521 | 3.458546 | 0.332024 |
| CERS3-AS1 | 0.00389 | 0.007986 | 0.087912 | 0.492848 | 0.316965 |
| CHL1-AS2 | 0.000293 | 0.000825 | 0.079416 | 0.327506 | 0.41968 |
| CISTR | 0.001816 | 0.004125 | 0.025092 | 0.152874 | 0.49468 |
| CTC-296K1.3 | 0.004419 | 0.008931 | 0.568655 | 1.553638 | 0.404354 |
| CTC-296K1.4 | 0.004858 | 0.009703 | 0.510565 | 1.356915 | 0.419081 |
| CTC-297N7.9 | 0.00036 | 0.00099 | 0.398673 | 1.128423 | 0.405946 |
| CTC-510F12.2 | 0.003133 | 0.006604 | 1.400342 | 3.440352 | 0.423783 |
| CTC-510F12.7 | 0.002971 | 0.006309 | 0.647131 | 2.052145 | 0.347156 |
| CTD-2089N3.1 | 0.000715 | 0.001804 | 0.80802 | 2.666641 | 0.328203 |
| CTD-2089N3.2 | 0.00067 | 0.001702 | 0.271949 | 1.347981 | 0.256874 |
| CTD-2314G24.2 | 0.007805 | 0.014662 | 0.404643 | 1.069436 | 0.431527 |
| DKFZp779M0652 | 1.65E-07 | 9.45E-07 | 0.165546 | 0.45553 | 0.478004 |
| FENDRR | 0.000513 | 0.001351 | 2.819012 | 6.906975 | 0.416587 |
| FGF14-AS2 | 0.000357 | 0.000983 | 0.571524 | 1.561545 | 0.404156 |
| FRMD6-AS2 | 0.000168 | 0.000506 | 0.081297 | 0.279388 | 0.477868 |
| GAS1RR | 0.000701 | 0.001773 | 0.101022 | 0.402867 | 0.399752 |
| HAND2-AS1 | 0.005919 | 0.011492 | 0.938004 | 5.734788 | 0.177899 |
| INAFM2 | 0.004949 | 0.009858 | 12.32729 | 51.76389 | 0.239614 |
| KB-68A7.1 | 0.003848 | 0.007912 | 0.357773 | 1.637706 | 0.263435 |
| LHX5-AS1 | 0.001803 | 0.0041 | 0.018891 | 0.351871 | 0.263109 |
| LINC00332 | 0.011821 | 0.020964 | 0.038671 | 0.181878 | 0.491952 |
| LINC00365 | 0.00269 | 0.005798 | 0.56079 | 2.110089 | 0.298988 |
| LINC00582 | 0.001303 | 0.003072 | 0.224165 | 0.846015 | 0.342663 |
| LINC00671 | 0.003089 | 0.006526 | 0.072132 | 0.339889 | 0.391307 |
| LINC00844 | 0.000681 | 0.001726 | 0.031889 | 0.215531 | 0.417992 |
| LINC00982 | 0.003736 | 0.00771 | 0.9429 | 3.674985 | 0.276266 |
| LINC01018 | 0.001383 | 0.003236 | 0.206394 | 0.622029 | 0.424352 |
| LINC01055 | 0.002873 | 0.006127 | 0.112641 | 0.415002 | 0.412893 |
| LINC01082 | 3.74E-05 | 0.00013 | 1.439265 | 4.830414 | 0.312198 |
| LINC01336 | 2.05E-06 | 9.42E-06 | 0.420524 | 1.922511 | 0.257365 |
| LINC01497 | 0.02195 | 0.036056 | 0.049787 | 0.274307 | 0.40017 |
| LINC01579 | 0.015418 | 0.026529 | 0.228797 | 0.634881 | 0.447415 |
| LL22NC03-75H12.2 | 0.016811 | 0.028559 | 0.204387 | 0.588409 | 0.44216 |
| LRRC3-AS1 | 0.003264 | 0.006845 | 0.076525 | 0.464346 | 0.312797 |
| MBNL1-AS1 | 0.011671 | 0.02075 | 1.277836 | 3.606294 | 0.371756 |
| MEF2C-AS1 | 3.54E-05 | 0.000123 | 0.081618 | 0.28145 | 0.476126 |
| MIR100HG | 0.00719 | 0.013634 | 2.182605 | 6.064029 | 0.370311 |
| MIR22HG | 2.19E-08 | 1.48E-07 | 4.916958 | 10.3142 | 0.481742 |
| MIR497HG | 0.000479 | 0.001271 | 0.16778 | 0.485418 | 0.457417 |
| MRGPRF-AS1 | 0.00129 | 0.003046 | 0.063236 | 0.26548 | 0.446635 |
| NALT1 | 0.00212 | 0.00472 | 0.674121 | 3.290002 | 0.228354 |
| NCOA7-AS1 | 0.006297 | 0.012117 | 0.135927 | 0.44401 | 0.433681 |
| PART1 | 0.000671 | 0.001704 | 0.41381 | 1.883197 | 0.259082 |
| PCAT18 | 0.00039 | 0.001064 | 0.104572 | 0.697909 | 0.256385 |
| PGM5-AS1 | 0.001987 | 0.00446 | 0.585009 | 5.662828 | 0.118867 |
| PWAR6 | 0.002832 | 0.006052 | 0.376347 | 1.401547 | 0.317238 |
| RBMS3-AS3 | 9.72E-05 | 0.000308 | 0.153404 | 0.615874 | 0.353978 |
| RP11-1060J15.4 | 0.014044 | 0.024419 | 0.674421 | 1.782155 | 0.411454 |
| RP11-1069G10.1 | 0.012109 | 0.0214 | 0.166328 | 0.674575 | 0.343838 |
| RP11-1101H11.1 | 0.000935 | 0.002289 | 0.126645 | 0.362201 | 0.49036 |
| RP11-116D2.1 | 0.028428 | 0.045194 | 0.071881 | 1.615001 | 0.100222 |
| RP11-126O1.6 | 0.009926 | 0.018054 | 0.31182 | 0.873026 | 0.423237 |
| RP11-131H24.4 | 0.007868 | 0.014765 | 0.445145 | 1.128437 | 0.443771 |
| RP11-148L24.1 | 0.002824 | 0.006041 | 0.118276 | 0.585717 | 0.318318 |
| RP11-15I11.3 | 0.012073 | 0.021355 | 0.409728 | 0.930366 | 0.494706 |
| RP11-161D15.1 | 0.021786 | 0.035807 | 0.121637 | 0.400293 | 0.443015 |
| RP11-195F19.9 | 0.005228 | 0.010322 | 0.976363 | 2.18723 | 0.470597 |
| RP11-209E8.1 | 0.015841 | 0.027147 | 0.088028 | 0.53911 | 0.294203 |
| RP11-21A7A.2 | 0.002665 | 0.005754 | 0.093477 | 0.478084 | 0.334687 |
| RP11-248N22.1 | 0.002276 | 0.005022 | 0.029576 | 0.21718 | 0.408524 |
| RP11-259K15.2 | 0.025769 | 0.04144 | 0.088192 | 0.376298 | 0.395115 |
| RP11-259O2.1 | 0.001056 | 0.002553 | 0.112962 | 0.383058 | 0.440861 |
| RP11-25K19.1 | 0.000133 | 0.000409 | 1.043949 | 2.382181 | 0.460865 |
| RP11-279O9.4 | 1.43E-06 | 6.85E-06 | 0.447953 | 1.042857 | 0.479459 |
| RP1-127D3.4 | 0.001056 | 0.002553 | 0.085643 | 0.436693 | 0.345902 |
| RP11-307B6.3 | 0.002462 | 0.005372 | 0.173326 | 0.792787 | 0.30615 |
| RP11-311F12.2 | 0.003761 | 0.007753 | 0.138812 | 0.577019 | 0.352741 |
| RP11-356J5.12 | 0.000637 | 0.001629 | 0.445766 | 1.042969 | 0.477499 |
| RP11-371M22.1 | 0.005326 | 0.010491 | 0.049098 | 0.468139 | 0.262432 |
| RP11-384F7.2 | 0.00032 | 0.000894 | 0.077981 | 0.368041 | 0.380268 |
| RP11-384P7.7 | 0.001504 | 0.003493 | 0.142842 | 0.408333 | 0.477722 |
| RP11-389G6.3 | 0.014889 | 0.025717 | 0.048303 | 0.395151 | 0.299511 |
| RP11-404E16.1 | 0.017934 | 0.030196 | 0.816036 | 1.826328 | 0.475535 |
| RP11-429A20.4 | 0.016996 | 0.028818 | 0.064516 | 0.31345 | 0.397911 |
| RP11-475B2.1 | 0.02125 | 0.035028 | 0.068957 | 0.298666 | 0.423806 |
| RP11-489D6.2 | 0.001987 | 0.00446 | 0.053421 | 0.337362 | 0.350788 |
| RP11-514D23.2 | 9.38E-05 | 0.000298 | 0.265178 | 0.73045 | 0.439736 |
| RP11-532F6.3 | 0.001809 | 0.00411 | 0.48212 | 1.602056 | 0.34201 |
| RP11-554A11.5 | 0.012878 | 0.022587 | 0.636989 | 1.77514 | 0.393031 |
| RP11-554A11.9 | 0.02158 | 0.035507 | 0.724855 | 2.866466 | 0.27806 |
| RP11-60A24.3 | 0.023027 | 0.037589 | 0.098906 | 0.301462 | 0.495454 |
| RP11-613D13.8 | 0.011902 | 0.021091 | 0.115569 | 0.519813 | 0.347798 |
| RP11-617F23.1 | 0.002385 | 0.005226 | 3.004435 | 7.189913 | 0.425853 |
| RP11-629G13.1 | 0.001723 | 0.003936 | 0.039797 | 0.182208 | 0.495368 |
| RP11-64B16.4 | 0.000767 | 0.001917 | 0.069758 | 0.263182 | 0.467417 |
| RP11-6O2.3 | 0.013304 | 0.023265 | 0.635677 | 2.847184 | 0.24962 |
| RP11-701H24.3 | 0.011137 | 0.019923 | 0.511364 | 1.148404 | 0.489716 |
| RP11-701H24.4 | 0.001184 | 0.002826 | 0.186145 | 0.520276 | 0.461319 |
| RP11-770J1.3 | 0.003976 | 0.008133 | 0.404644 | 1.098151 | 0.421186 |
| RP11-776A13.1 | 0.004614 | 0.00928 | 0.112222 | 0.34405 | 0.477924 |
| RP11-798M19.6 | 0.002256 | 0.004984 | 0.525974 | 1.373795 | 0.424736 |
| RP11-864I4.4 | 0.000565 | 0.001469 | 0.287776 | 0.70812 | 0.47985 |
| RP11-867G23.10 | 0.000246 | 0.00071 | 0.365427 | 2.83926 | 0.158348 |
| RP11-875O11.1 | 0.000115 | 0.000358 | 0.353708 | 0.844652 | 0.480291 |
| RP11-887P2.5 | 0.005757 | 0.011217 | 0.17108 | 0.56679 | 0.406545 |
| RP1-193H18.3 | 0.010848 | 0.019477 | 0.163765 | 0.53731 | 0.413873 |
| RP11-963H4.3 | 0.015068 | 0.025991 | 0.169036 | 0.492109 | 0.454369 |
| RP5-1024C24.1 | 0.003942 | 0.008072 | 0.055343 | 0.238913 | 0.458357 |
| RP5-1125N11.2 | 0.009623 | 0.017579 | 0.198912 | 0.583953 | 0.437035 |
| SEMA3B-AS1 | 0.021956 | 0.036061 | 1.02874 | 2.458021 | 0.441255 |
| SERTAD4-AS1 | 0.009013 | 0.016597 | 3.179702 | 7.646536 | 0.423377 |
| SNHG14 | 0.002162 | 0.004802 | 0.436096 | 1.106172 | 0.44446 |
| SOX21-AS1 | 0.000601 | 0.001549 | 1.325735 | 5.351445 | 0.261533 |
| TMEM220-AS1 | 5.90E-05 | 0.000196 | 0.483054 | 1.483815 | 0.368133 |
| TTTY14 | 0.003958 | 0.008099 | 0.4105 | 0.930015 | 0.495624 |
| UBXN10-AS1 | 0.000906 | 0.002226 | 0.978609 | 6.199624 | 0.171218 |
| UG0898H09 | 0.005087 | 0.010101 | 0.09068 | 0.321772 | 0.452092 |
| UNC5B-AS1 | 0.001417 | 0.003306 | 1.533461 | 4.00591 | 0.397832 |
| ZNF667-AS1 | 0.000606 | 0.001562 | 1.295529 | 3.001956 | 0.449887 |
