# Supplementary material for: Function and regulation annotation of up‐regulated long non‐coding RNA LINC01234 in gastric cancer
Source: J Clin Lab Anal. 2020 Feb 3;34(5):e23210. doi: 10.1002/jcla.23210 (PMC7246363; doi:10.1002/jcla.23210)
Supplement: Supplementary file 3 [file JCLA-34-e23210-s003.docx]

**Supplementary Table 3**

| **Gene1** | **Gene2** | **Regulation Type** |
| --- | --- | --- |
| LINC01234 | hsa-miR-100-5p | miRNA |
| LINC01234 | hsa-miR-1207-5p | miRNA |
| LINC01234 | hsa-miR-122-5p | miRNA |
| LINC01234 | hsa-miR-1236-3p | miRNA |
| LINC01234 | hsa-miR-124-3p | miRNA |
| LINC01234 | hsa-miR-125a-3p | miRNA |
| LINC01234 | hsa-miR-125a-5p | miRNA |
| LINC01234 | hsa-miR-1284 | miRNA |
| LINC01234 | hsa-miR-129-1-3p | miRNA |
| LINC01234 | hsa-miR-129-2-3p | miRNA |
| LINC01234 | hsa-miR-1296-5p | miRNA |
| LINC01234 | hsa-miR-1297 | miRNA |
| LINC01234 | hsa-miR-137 | miRNA |
| LINC01234 | hsa-miR-143-3p | miRNA |
| LINC01234 | hsa-miR-145-5p | miRNA |
| LINC01234 | hsa-miR-146a-5p | miRNA |
| LINC01234 | hsa-miR-149-3p | miRNA |
| LINC01234 | hsa-miR-15a-3p | miRNA |
| LINC01234 | hsa-miR-200c-3p | miRNA |
| LINC01234 | hsa-miR-206 | miRNA |
| LINC01234 | hsa-miR-26b-5p | miRNA |
| LINC01234 | hsa-miR-28-5p | miRNA |
| LINC01234 | hsa-miR-29a-3p | miRNA |
| LINC01234 | hsa-miR-302b-3p | miRNA |
| LINC01234 | hsa-miR-3174 | miRNA |
| LINC01234 | hsa-miR-320a | miRNA |
| LINC01234 | hsa-miR-335-5p | miRNA |
| LINC01234 | hsa-miR-338-3p | miRNA |
| LINC01234 | hsa-miR-361-5p | miRNA |
| LINC01234 | hsa-miR-409-3p | miRNA |
| LINC01234 | hsa-miR-429 | miRNA |
| LINC01234 | hsa-miR-4455 | miRNA |
| LINC01234 | hsa-miR-449a | miRNA |
| LINC01234 | hsa-miR-484 | miRNA |
| LINC01234 | hsa-miR-485-5p | miRNA |
| LINC01234 | hsa-miR-490-3p | miRNA |
| LINC01234 | hsa-miR-491-5p | miRNA |
| LINC01234 | hsa-miR-508-5p | miRNA |
| LINC01234 | hsa-miR-519d-3p | miRNA |
| LINC01234 | hsa-miR-520d-3p | miRNA |
| LINC01234 | hsa-miR-524-5p | miRNA |
| LINC01234 | hsa-miR-532-5p | miRNA |
| LINC01234 | hsa-miR-542-3p | miRNA |
| LINC01234 | hsa-miR-584-3p | miRNA |
| LINC01234 | hsa-miR-613 | miRNA |
| LINC01234 | hsa-miR-647 | miRNA |
| LINC01234 | hsa-miR-802 | miRNA |
| LINC01234 | hsa-miR-873-5p | miRNA |
| LINC01234 | hsa-miR-940 | miRNA |
| LINC01234 | ADNP | TF |
| LINC01234 | CHAMP1 | TF |
| LINC01234 | E2F5 | TF |
| LINC01234 | ELK1 | TF |
| LINC01234 | ETV4 | TF |
| LINC01234 | FOXK2 | TF |
| LINC01234 | HDAC1 | TF |
| LINC01234 | HDAC2 | TF |
| LINC01234 | IRF3 | TF |
| LINC01234 | KDM1A | TF |
| LINC01234 | MCM7 | TF |
| LINC01234 | MYBL2 | TF |
| LINC01234 | NBN | TF |
| LINC01234 | NFE2 | TF |
| LINC01234 | NFYA | TF |
| LINC01234 | PHF8 | TF |
| LINC01234 | POLR2H | TF |
| LINC01234 | RAD51 | TF |
| LINC01234 | SMARCA4 | TF |
| LINC01234 | SREBF1 | TF |
| LINC01234 | TAF1 | TF |
| LINC01234 | TFAP4 | TF |
| LINC01234 | ZBTB11 | TF |
| LINC01234 | ZBTB33 | TF |
| LINC01234 | ZKSCAN1 | TF |
| LINC01234 | ZNF664 | TF |
| LINC01234 | FOS | TF |
| LINC01234 | JUN | TF |
| LINC01234 | JUNB | TF |
| LINC01234 | JUND | TF |
| LINC01234 | NR4A1 | TF |
| LINC01234 | ADAT1 | RBP |
| LINC01234 | AGFG1 | RBP |
| LINC01234 | AGO2 | RBP |
| LINC01234 | AQR | RBP |
| LINC01234 | BARD1 | RBP |
| LINC01234 | BICC1 | RBP |
| LINC01234 | BOLL | RBP |
| LINC01234 | BRCA1 | RBP |
| LINC01234 | CDC5L | RBP |
| LINC01234 | CHTOP | RBP |
| LINC01234 | CNOT6 | RBP |
| LINC01234 | CPSF1 | RBP |
| LINC01234 | CPSF2 | RBP |
| LINC01234 | CPSF3 | RBP |
| LINC01234 | CPSF4 | RBP |
| LINC01234 | CPSF6 | RBP |
| LINC01234 | CSTF1 | RBP |
| LINC01234 | CSTF2 | RBP |
| LINC01234 | CSTF3 | RBP |
| LINC01234 | DCP2 | RBP |
| LINC01234 | DDX10 | RBP |
| LINC01234 | DDX11 | RBP |
| LINC01234 | DDX18 | RBP |
| LINC01234 | DDX21 | RBP |
| LINC01234 | DDX31 | RBP |
| LINC01234 | DDX43 | RBP |
| LINC01234 | DDX46 | RBP |
| LINC01234 | DDX47 | RBP |
| LINC01234 | DDX52 | RBP |
| LINC01234 | DDX53 | RBP |
| LINC01234 | DDX54 | RBP |
| LINC01234 | DDX55 | RBP |
| LINC01234 | DDX56 | RBP |
| LINC01234 | DHX34 | RBP |
| LINC01234 | DHX8 | RBP |
| LINC01234 | DKC1 | RBP |
| LINC01234 | DPPA5 | RBP |
| LINC01234 | EIF3B | RBP |
| LINC01234 | ERAL1 | RBP |
| LINC01234 | FMR1 | RBP |
| LINC01234 | FUBP1 | RBP |
| LINC01234 | FUS | RBP |
| LINC01234 | FXR1 | RBP |
| LINC01234 | G3BP1 | RBP |
| LINC01234 | GRB7 | RBP |
| LINC01234 | HELZ2 | RBP |
| LINC01234 | ILF2 | RBP |
| LINC01234 | ILF3 | RBP |
| LINC01234 | IREB2 | RBP |
| LINC01234 | KHDC1 | RBP |
| LINC01234 | LARP4 | RBP |
| LINC01234 | LSM5 | RBP |
| LINC01234 | LSM7 | RBP |
| LINC01234 | MEX3A | RBP |
| LINC01234 | MEX3B | RBP |
| LINC01234 | MEX3D | RBP |
| LINC01234 | MOV10 | RBP |
| LINC01234 | MRM1 | RBP |
| LINC01234 | NCBP1 | RBP |
| LINC01234 | NIP7 | RBP |
| LINC01234 | NOL6 | RBP |
| LINC01234 | NOL8 | RBP |
| LINC01234 | NONO | RBP |
| LINC01234 | NOP2 | RBP |
| LINC01234 | NOP56 | RBP |
| LINC01234 | NSUN6 | RBP |
| LINC01234 | NXF3 | RBP |
| LINC01234 | OAS2 | RBP |
| LINC01234 | OAS3 | RBP |
| LINC01234 | OOEP | RBP |
| LINC01234 | PA2G4 | RBP |
| LINC01234 | PAIP1 | RBP |
| LINC01234 | PARN | RBP |
| LINC01234 | PATL1 | RBP |
| LINC01234 | PATL2 | RBP |
| LINC01234 | PNPT1 | RBP |
| LINC01234 | PPRC1 | RBP |
| LINC01234 | PSPC1 | RBP |
| LINC01234 | PTBP1 | RBP |
| LINC01234 | PTBP3 | RBP |
| LINC01234 | PUM1 | RBP |
| LINC01234 | PUM3 | RBP |
| LINC01234 | PUS7 | RBP |
| LINC01234 | PUS7L | RBP |
| LINC01234 | PUSL1 | RBP |
| LINC01234 | RBM12 | RBP |
| LINC01234 | RBM14 | RBP |
| LINC01234 | RBM15 | RBP |
| LINC01234 | RBM19 | RBP |
| LINC01234 | RBM26 | RBP |
| LINC01234 | RBM28 | RBP |
| LINC01234 | RBM34 | RBP |
| LINC01234 | RBM39 | RBP |
| LINC01234 | RBM41 | RBP |
| LINC01234 | RBM46 | RBP |
| LINC01234 | RBM48 | RBP |
| LINC01234 | RBMS2 | RBP |
| LINC01234 | RBMX2 | RBP |
| LINC01234 | RDM1 | RBP |
| LINC01234 | RNPS1 | RBP |
| LINC01234 | RPP25 | RBP |
| LINC01234 | SARNP | RBP |
| LINC01234 | SART3 | RBP |
| LINC01234 | SF3B4 | RBP |
| LINC01234 | SFPQ | RBP |
| LINC01234 | SNRPA | RBP |
| LINC01234 | SRRM1 | RBP |
| LINC01234 | SRSF1 | RBP |
| LINC01234 | SRSF2 | RBP |
| LINC01234 | SRSF6 | RBP |
| LINC01234 | STAU1 | RBP |
| LINC01234 | SUGP2 | RBP |
| LINC01234 | SURF6 | RBP |
| LINC01234 | SYNJ2 | RBP |
| LINC01234 | TDRKH | RBP |
| LINC01234 | TERT | RBP |
| LINC01234 | THOC1 | RBP |
| LINC01234 | THOC2 | RBP |
| LINC01234 | THOC6 | RBP |
| LINC01234 | TLR8 | RBP |
| LINC01234 | TRNT1 | RBP |
| LINC01234 | TRUB1 | RBP |
| LINC01234 | TSN | RBP |
| LINC01234 | UBR5 | RBP |
| LINC01234 | UHMK1 | RBP |
| LINC01234 | WT1 | RBP |
| LINC01234 | XPO1 | RBP |
| LINC01234 | XPO5 | RBP |
| LINC01234 | XRN1 | RBP |
| LINC01234 | ZC3H8 | RBP |
| LINC01234 | ZNF74 | RBP |
| LINC01234 | CELF2 | RBP |
| LINC01234 | LARP6 | RBP |
| LINC01234 | MYEF2 | RBP |
| LINC01234 | NOVA1 | RBP |
| LINC01234 | RBM20 | RBP |
| LINC01234 | RBM24 | RBP |
| LINC01234 | RBPMS | RBP |
| Gene1 | Gene2 | Regulation Type |
