# Supplementary material for: Function and regulation annotation of up‐regulated long non‐coding RNA LINC01234 in gastric cancer
Source: J Clin Lab Anal. 2020 Feb 3;34(5):e23210. doi: 10.1002/jcla.23210 (PMC7246363; doi:10.1002/jcla.23210)
Supplement: Supplementary file 4 [file JCLA-34-e23210-s004.docx]

**Supplementary Table 4**

| **Gene1** | **Gene2** | **Regulation Type** | **Number of datasets that observe co-expression** |
| --- | --- | --- | --- |
| LINC01234 | ELK1 | TF | 3 |
| LINC01234 | ZNF664 | TF | 3 |
| LINC01234 | CPSF3 | RBP | 3 |
| LINC01234 | DDX18 | RBP | 6 |
| LINC01234 | DKC1 | RBP | 3 |
| LINC01234 | FUS | RBP | 4 |
| LINC01234 | KHDC1 | RBP | 4 |
| LINC01234 | LSM5 | RBP | 3 |
| LINC01234 | MEX3B | RBP | 6 |
| LINC01234 | NOP2 | RBP | 7 |
| LINC01234 | NOP56 | RBP | 8 |
| LINC01234 | PA2G4 | RBP | 8 |
| LINC01234 | PARN | RBP | 3 |
| LINC01234 | PATL1 | RBP | 3 |
| LINC01234 | PNPT1 | RBP | 3 |
| LINC01234 | PUS7 | RBP | 3 |
| LINC01234 | RBM19 | RBP | 8 |
